# Identification of a novel Sorcin isoform with a different C-terminal but intact dimerization property

**DOI:** 10.1038/s41598-023-40913-z

**Published:** 2023-09-14

**Authors:** Supriya Tanwar, Faizan Abul Qais, Farheen Naaz, Naira Rashid, Faizan Ahmad, Sayeed ur Rehman

**Affiliations:** 1 Department of Biochemistry, School of Chemical and Life Sciences, Jamia Hamdard, New Delhi, 110062 India; 2 Department of Agricultural Microbiology, Faculty of Agricultural Sciences, Aligarh Muslim University, Aligarh, UP 202002 India

**Keywords:** Cancer, Computational biology and bioinformatics, Molecular biology

## Abstract

Sorcin (Sri), a member of penta EF-hand protein family plays a diverse role in maintaining calcium homeostasis, cell cycle and vesicular trafficking. Sri is highly conserved amongst mammals and consists of N-terminal glycine rich domain and C-terminal calcium binding domain that mediates its dimerization and interacts with different compounds. In the present study, with the help of combination of computational and molecular biology techniques, we have identified a novel isoform (Sri-N) in mouse which differs only in the C-terminal domain with that of Sri reported earlier. The novel isoform contains a new last exon that is different from the one present in the reported transcript (Sri). The presence of the novel isoform was further validated in different tissues by RT-PCR and DNA sequencing. The transcript was conceptually translated and subjected to in-silico analysis using different bioinformatics tools. The novel transcript variant encodes for a longer protein isoform without any change in the sub-cellular localization as predicted by PSORT-II online tool. Molecular modelling was performed to compare the structural changes in Sri-N and Sri isoforms. The structural characterization of the novel isoform using MD simulation depicted its overall stability under the physiological conditions. The molecular docking of proteins with various chemotherapeutic drugs revealed that their binding affinity is more for Sri-N as compared to that for the previously reported transcript Sri.

## Introduction

Sri (soluble resistance-related calcium-binding protein) is a highly conserved protein amongst vertebrates and is ubiquitously expressed in mammals. It is a member of a penta EF-hand (PEF) protein family that plays a versatile role in maintaining calcium homeostasis, cell cycle and vesicular traffic^1^. Its multifaceted role has also been discerned in non-neoplastic disorders like diabetes, viral infection, infertility and disorders associated with nervous system^2^. Sri has also been established to play a cardinal role in neoplastic disorders like various types of cancers especially where the malignancies have turned multidrug-resistant^3^. Sri is one of the highly expressed calcium binding proteins in humans. It undergoes conformational changes upon calcium binding thereby allowing its translocation from cytoplasm to membrane where it targets a number of signaling cascades^4^. Sri is ubiquitously expressed in various tissues including lymphocytes, monocytes, kidney, brain, skeletal muscle, cardiac muscle, liver, breast and skin, comprising about 3% of the vertebrate’s proteome^5^. Due to its pervasive expression in different tissues, it is likely to be involved in several cellular processes such as mitotic progression and cytokinesis, apoptosis, cell death and cardiac muscle contraction^6^.

**Figure 1:**
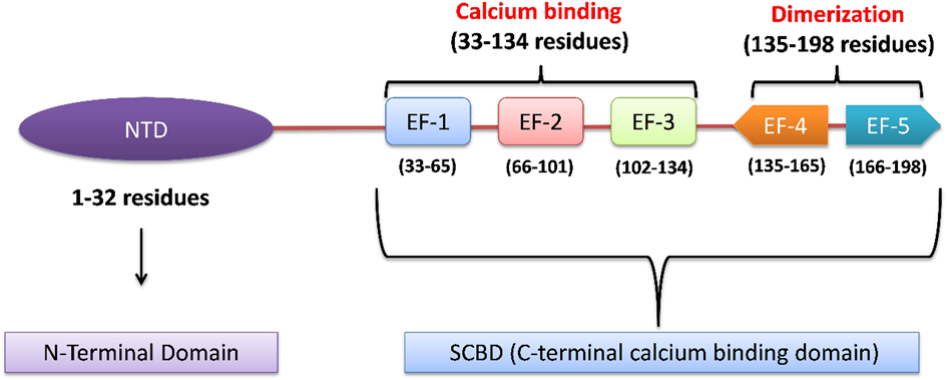
Structural domains of the mouse Sorcin (Sri) gene. Sri is composed of 198 amino acids and contains N-terminal domain of 32 residues (1–32) and C-terminal domain of 166 residues (33–198) which is subdivided into calcium binding domain (33–134) and dimerization domain (135–198).

From structural standpoint, Sri acts as a homodimer in the absence of calcium. Each monomer comprises of two domains, namely, glycine rich N-terminal domain and a Ca^2+^ binding C-terminal domain (SCBD) with 5 EF hands (Fig. 1). The EF hands are structural motifs containing helix-loop-helix elements. Furthermore, the SCBD is subdivided into 2 subcategories, i.e., the EF1- EF3 containing subdomain that shows high affinity towards calcium, while the subdomain consisting of EF4-EF5 contains several possible phosphorylation sites and mediates dimerization^7^. In general, calcium binds to EF1-EF3 hands that bring about a large conformational change in Sri structure, involving a movement of D-helix that exposes its hydrophobic residues, EF loop and G-helix to the solvent subsequently reducing its solubility and facilitating its translocation from cytosol to plasma membrane where it binds and regulates a series of target proteins^8^.

Alternative splicing is a major gene expression mechanism in eukaryotic genome that allows a single gene to produce multiple transcripts in about 95% of human genes and gives rise to structurally discrete mRNA and protein isoforms^9^. This post transcriptional phenomenon of generating protein diversity has been established in Sri of both humans and mouse^10^. Human Sri gene is located on chromosome number 7 and spans 23 kb of human genome^11^. It has been reported to have at least 4 different isoforms in human, i.e., isoform A, B, C and D differing in their 5ʹ UTR regions lacking part of N-terminal domain or amino acids of the C-terminal domain. While in mouse, Sri gene is present on Chromosome number 5 and spans approximately 23.2 kb of mouse genome. Moreover, 2 alternatively spliced isoforms are reported in mouse, both differ in their 5ʹ coding region. As per the Mouse Genome Informatics (MGI) database, the large transcript, i.e., isoform 1 codes for a 198-amino acid residue long protein (Accession No: NM_001080974.2) while the smaller isoform 2 codes for 183-amino acid residue long protein (Accession No: NM_025618.3).

Due to the functional role of Sri in controlling calcium homeostasis and maintenance of cellular processes, we have studied the alternative splicing of Sri in mouse. In this study, a combination of bioinformatics tools and molecular biology approach has been successfully incorporated to identify a novel alternatively spliced isoform of mouse Sri Gene. The predicted alternatively spliced variant differs from the existing isoforms in their C-terminal domain. In addition, the subcellular localization, post transcriptional modifications, translational efficacy and half-life of the conceptually translated protein sequence were further studied with the help of different computational tools.

## Materials and methods

### Computational prediction of novel exon

For the identification of alternatively spliced novel variants of mouse Sri gene, a combination of different bioinformatics tools were used to predict novel exons. The genomic DNA, cDNA and protein sequences were retrieved from Mouse Genome Informatics (MGI) at http://www.informatics.jax.org/. The ± 20 kb genomic sequence was analyzed to predict genes using online tool FGENESH available at http://www.softberry.com/berry.phtml. Reported exons were located on the genomic sequence and 5ʹ UTR, intronic sequence and 3ʹ UTR were examined using exon finding tool, FEX, available at http://www.softberry.com/berry.phtml. Exons were selected based on their FEX score, and false positives were filtered out through manual curation. The coding nature of the predicted exons were studied using test code analysis (sequence manipulation suite). Exon specific primers were designed using OligoCalc and synthesized commercially (IDT Gemological Laboratories Worldwide). The sequences of the primers used for amplification of the transcript are given below:

- F1C: 5ʹ-TGG ACA AAC TCA GGA TCC GCT ATA-3ʹ (from Exon 1);
- F2C: 5ʹ-CTT TGC TGC TGT GGC TGG ACA G-3ʹ (from Exon 2);
- E8’R: 5ʹ-TCA TAA TCT TGG CAT CGA TAG GGC-3ʹ (from Exon 8);
- R1: 5ʹ-TAG ACG GTC ATG ACA CAC TGG ATG-3ʹ (from Exon 7).

### First strand cDNA synthesis

Samples of total RNA from mouse pancreas (Cat# R1334188-50), adipose tissue (Cat# R1334003-50), and liver (Cat# R1334149) were obtained from BioChain, USA. The mRNA from these mouse tissues were used as template for cDNA synthesis using RevertAid H Minus First Strand cDNA Synthesis Kit **(**Thermo Scientific, USA) following the manufacturer’s protocol.

### Touchdown and semi-nested PCR

To validate the existence of predicted transcripts, touchdown PCR was performed using first strand cDNA as templates and exon specific primers. Amplification was done using the following conditions: Initial denaturation for 3 min at 95 °C followed by 34 cycles of denaturation for 30 s at 95 °C and annealing for 30 s at 66.6 °C with a decrease of 0.2 °C per cycle and extension for 1 min at 72 °C followed by final extension at 72 °C for 8 min^12^. The PCR product obtained was further used as template in the subsequent semi-nested PCR using one of the primers from internal position. To amplify the reported transcript, forward primer F1C was used along with R1 as reverse primer in touchdown PCR followed by semi-nested PCR using the same reverse primer but with an internal forward primer, F2C. The novel transcript was amplified using F1C as forward and E8'R as reverse primers while F2C forward primer was used as an internal primer in semi-nested reaction. The product obtained after semi-nested PCR was then subjected to electrophoresis using 1.2% agarose gel and stained with ethidium bromide and further visualized on UV trans-illuminator (MaestroGen, Taiwan)**.**

### DNA sequencing

To confirm the sequence of the PCR products, gel extraction was performed using QIAquick Gel Extraction Kit (Qiagen, Hilden, Germany) and purified DNA was outsourced to a commercial sequencing facility (Macrogen, South Korea) for sanger sequencing using exon specific primers.

### Computational analysis to characterize the novel isoform

The sequence obtained after DNA sequencing was analyzed for the similarity and homology search with respect to the already reported isoform using non-redundant database of BLASTN and BLASTP (https://www.ncbi.nlm.nih.gov/blast/)^13^. After sequence confirmation, the novel sequence was conceptually translated using ExPaSy translate tool (https://web.expasy.org/translate/). Multiple sequence alignment of the protein sequences of both the reported (Sri) and novel (Sri-N) isoform was carried out using Clustal Omega (https://www.genome.jp/tools/clustalw/). The prediction of different physical and chemical parameters of the reported (Sri) and novel (Sri-N) isoforms was compared using ProtParam tool available at ExPaSy portal (https://web.expasy.org/protparam/). The sub-cellular localization of novel isoform was predicted using Psort-II server (https://psort.hgc.jp/form2.html)^14^.

### Homology modeling and molecular dynamics (MD) simulation

The structure of the novel protein was predicted using I-TASSER (http://zhanglab.ccmb.med.umich.edu/I-TASSER/) and the PDB ID: 1JUO (crystal structure of calcium free human Sri) was taken as reference protein for homology modeling. The pairwise sequence alignment was studied to check the identity and similarity of both the isoforms using EMBOSS Needle tool (https://www.ebi.ac.uk/Tools/psa/emboss_needle/). The crystal structure of 1JUO lacks first 26 amino acids from the N-terminal and therefore these amino acids were not taken for homology modeling in case of Sri-N. We submitted the amino acid sequences from 27 to 201 to I-TASSER server, and the model with best I-TASSER score was selected for further analysis. The models were subsequently elucidated based on sequence identity with high C-score, TM-score and highest resolution. The model obtained from I-TASSER was chosen and further studied by mimicking the actual physiological conditions (1 bar, 310 K and 0.15 M NaCl) to study their stability and dynamics. MD simulations of both Sri and Sri-N were carried out using GROMACS-2018.1 packages with amber99sb-ILDN force field^15,16^. The solvation step was carried out using TIP3P water model in a triclinic box followed by system neutralization by adding the counter ion, i.e., sodium or chlorine. The energy minimization of both systems was performed using the steepest descent minimization up to 50,000 steps in order to remove the weak van der Waals contacts. The systems were then equilibrated for 1 ns for both NVT and NPT to ensemble conditions such as constant pressure and constant volume for each protein. A 100 ns of standard MD simulation was performed for both proteins (Sri and Sri-N) in water and co-ordinates were simultaneously saved for the whole simulation period. Prior to analysis, the system was subjected to PBC (periodic boundary conditions) corrections. The root-mean-square deviations (RMSD), root-mean-square fluctuation (RMSF) and radius of gyration (*R*_g_) for both Sri and Sri-N were compared using GROMACS utility. Each system was run in triplicates.

### Dimerization study using protein–protein docking and MD simulation

The ClusPro server was used to predict the possible dimeric structures of both Sri and Sri-N proteins. First, the method was validated by docking the two monomers of Sri using ClusPro^17^. The docked dimer was found to be similar with the earlier reported structure. All models of the monomers of Sri and Sri-N were submitted to ClusPro and the following structures were obtained: (Sri)–(Sri) homodimer, (Sri-N)–(Sri-N) homodimer, (Sri)–(Sri-N) heterodimer. For the homodimerization, only single chain was given as input. For the heterodimerization, one chain of each isoform was given as input. Structures with lowest binding energy were further used for MD simulation and analysis studies.

### Protein–ligand docking

Molecular docking provides a valuable tool to study the interactions between small molecule and proteins and elucidate the binding behavior of the small molecule with different sites present on the proteins. It has been reported by computational studies that Sri might play a significant role in multi drug resistance (MDR) in human cancers against different chemotherapeutic drugs such as doxorubicin, etoposide, omacetaxine and vincristine^18,19^. Therefore, we intend to compare the interaction of small molecules with the reported Sri and novel isoform of Sri. The molecular docking was performed using AutoDock Vina^20^. During molecular docking, proteins were considered as rigid molecule and ligands as flexible molecules. The protein structure was prepared in AutoDockTools-1.5.6 by deleting water molecules and adding polar hydrogen and Kollman’s charge using edit option. A grid box was made around the protein using the AutoDock Tools, the spacing of grid box was set to 1 Å and the size of the grid box was 48 × 48 × 48 with centre as x = 73.012, y = 101.053, z = 9.055. The target proteins were finally saved in PDBQT format. 3D structures of all the drugs were retrieved from PubChem (www.pubchem.ncbi.nlm.nih.gov) in SDF format and converted to PDB format using chimera 1.14 software (California, SF, USA). The PDB file was further used for molecular docking studies. Within the AutoDock tools, ligands were loaded using ligand option and were made flexible by detecting roots to get the best possible conformations and ligands were saved in PDBQT format. The analysis of molecular docking was performed using Discovery Studio, AutoDock tools and PyMOL.

## Results

### In-silico prediction of alternatively spliced novel isoform of mouse Sri gene

**Figure 2:**
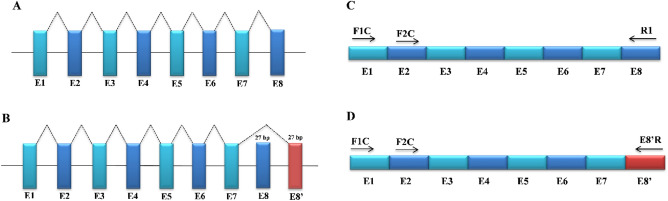
Schematic representation of genetic organization of the mouse Sri gene. (**A**) Splicing pattern of the reported transcript of Sri gene: The colored rectangular boxes E1–E8 refer to the exons and the solid interconnecting lines as introns. The dashed puckered lines show the splicing pattern of the reported exons. (**B**) The splicing pattern of the novel transcript (Sri-N): the dashed puckered line shows the new splicing pattern that generates a novel transcript Sri-N. The novel transcript is formed via splicing of exon-E7 with exon-E8ʹ and removing exon-E8 from the mature transcript. (**C**) Location of control primers designed for RT-PCR: The positive controls F1C and F2C were designed from the reported exons 1 and 2 along with the reverse primer R1 from exon 8. The black arrows depict the direction of the forward (FP) and reverse (RP) primers. (**D**) Primer designing from novel transcript for RT-PCR: F1C and F2C were taken from exons 1and 2, the reverse primer E8ʹR was obtained from the new exon (E8ʹ) of the novel transcript Sri-N.

The mouse Sri spans about 23.2 kb of mouse genome and is reported to have 2 alternatively spliced transcripts, isoform 1 (a primary transcript of 8 exons transcribed into a long protein of 198 amino acids) and isoform 2 (shorter variant, differing in the 5ʹ UTR and the 5ʹ coding region yielding a shorter protein of 183 amino acids) (Fig. 2A). Using a combination of different in silico tools and manual curation, we predicted one new coding exon named E8ʹ, positioned 1.16 kb downstream of exon E7 (Fig. 2B). This new exon was predicted to splice out the exon E8 and participate in alternative splicing with exon E7, giving rise to a new transcript that differs at their 3ʹ end. The sequences similarity of the novel exon was done using nucleotide sequence in an attempt to search for the available ESTs with the help of BLAST and none of them yielded any positive hits. Primers were designed from the regions specific to reported and novel exons as shown in Fig. 2C,D.

### Validation of predicted novel exon using RT-PCR

**Figure 3:**
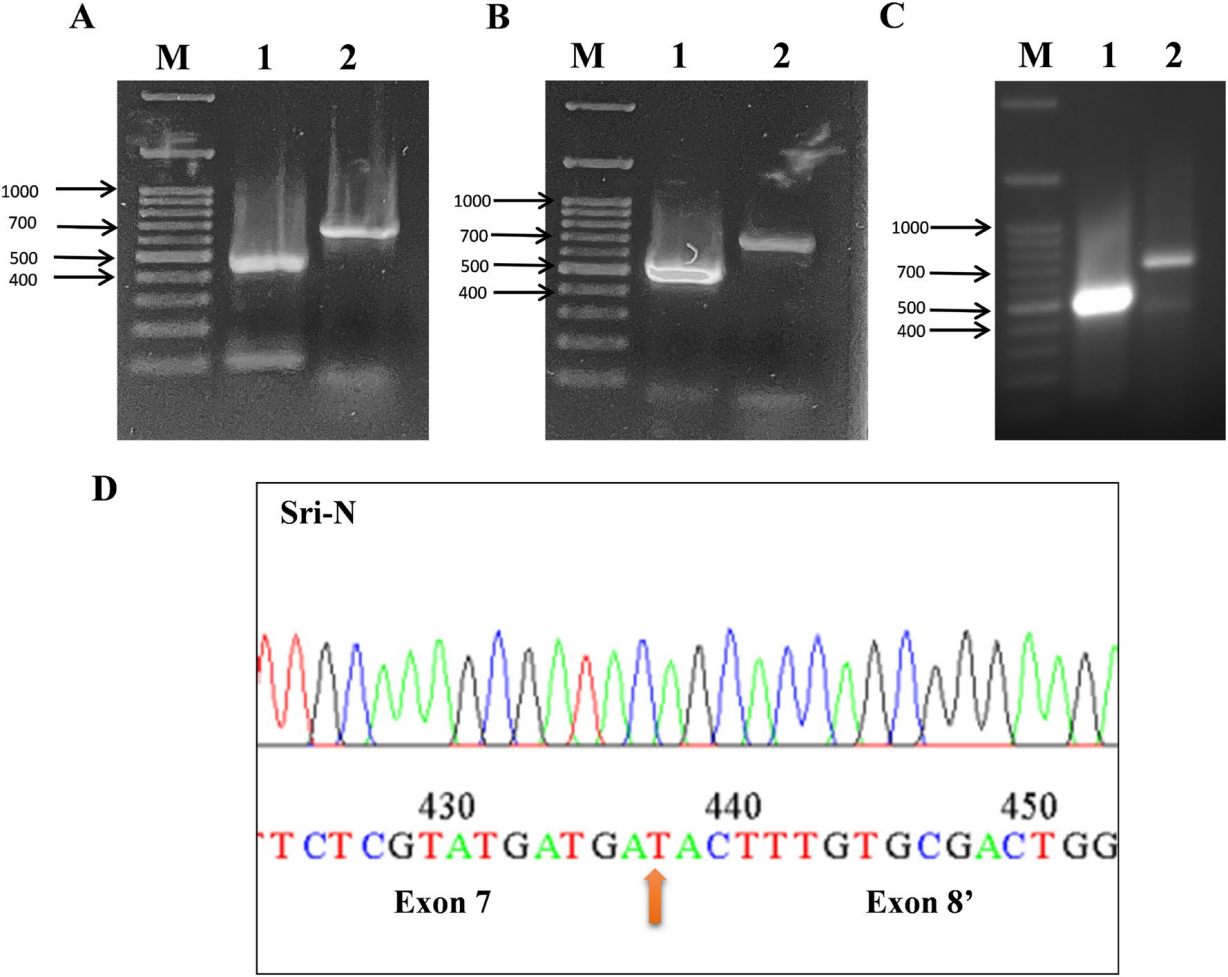
Agarose gel electrophoresis of semi-nested PCR products and DNA sequencing results. (**A**) RT-PCR amplification of both transcripts (Sri and Sri-N) using pancreas cDNA: The left side arrows indicate the size of the marker. Lane 1 with a band size of 484 bp corresponds to the reported isoform and served as positive control while lane 2 represents the Sri-N isoform having an amplicon size of approximately 650 bp. (**B**) RT-PCR experiment using adipose tissue cDNA: Lane M signifies marker while lane 1 and lane 2 represent the expression of Sri and Sri-N, respectively. The size of the amplicons was same as found in pancreas cDNA. (**C**) Expression of Sri and Sri-N in liver. Lane M signifies marker while lane 1 and lane 2 represent the expression of Sri and Sri-N, respectively. (**D**) The chromatogram represents the DNA sequencing results where the upward arrow denotes the junction between exon 7 and exon E8ʹ.

To validate the existence of novel transcript of Sri gene in the mouse tissues, RT-PCR was performed using transcript specific primers. Total RNA from different mouse tissues were obtained commercially and were reverse transcribed using oligo (dT)_18_ to attain tissue specific cDNA. This cDNA was then taken as a template for touchdown PCR using transcript specific forward primer F1C from exon 1 and reverse primer E8'R from novel exon E8ʹ. Forward primer F1C from exon 1 and reverse primer R1 from exon 8 were used to amplify reported transcript and served as a positive control for our experiments. The amplified PCR product from touchdown PCR was used as template in the subsequent semi-nested PCR. During the semi-nested reaction, primer F2C was used as an internal forward primer in both the reactions. The amplified PCR product from different tissues was fractioned by agarose gel electrophoresis. The size of the novel amplicon was found to be of expected size, approximately 650 bp in different tissues as seen in Fig. 3A,B. Lane 1 represents reported transcript (Sri), serving as positive control, and lane 2 represents the novel transcript (Sri-N) of expected size thus confirming the existence of a novel transcript in the tissues studied (pancreas and adipose). PCR product was excised from the gel and was subjected to gel purification followed by DNA sequencing. Sanger sequencing confirmed the identity of the novel transcript (Fig. 3C). The novel isoform was named as Sri-N and the sequence was submitted to NCBI database and is available online with the accession number OQ789964.

### In-silico analysis of novel isoforms of Sri

**Figure 4:**
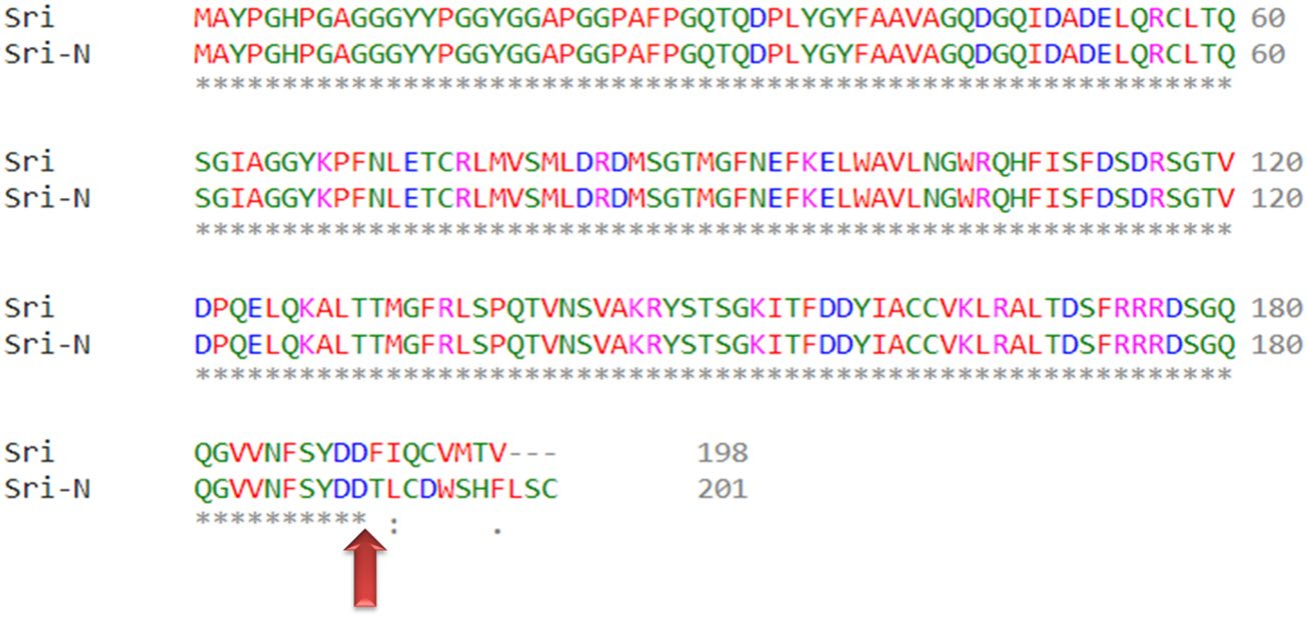
Sequences of the reported (Sri) and novel (Sri-N) isoforms. Alignment (Clustal omega) of amino acid sequences of the reported (Sri) and novel (Sri-N) isoforms. The names of the isoform are shown adjacent to the sequence. Each asterisk (*) shows position of the identical residues whereas the red upward arrow depicts the difference from Exon-E7 onwards.

In-silico analysis was carried out for comparative post-translational studies using conceptually translated sequence obtained by ExPASy translate tool. Difference can be seen in number and nature of encoded amino acid residues between the Sri and Sri-N isoforms (Table 1). The newly identified isoform Sri-N has 201 amino acids whereas the known Sri isoform is slightly smaller with 198 amino acids. Multiple sequence alignment using Clustal Omega shows the similarities and differences in the amino acid sequences of Sri and Sri-N (Fig. 4). The change in the relative molecular mass of Sri and Sri-N was found to vary from 21,627.26 to 21,998.57 Da and pI was found to be 5.32 and 5.29, respectively. Furthermore, the sub-cellular localization was predicted by using PSORT-II tool and as seen in Table 2, no significant differences was found in the cellular localization of Sri and Sri-N.

**Table 1:** Comparative sequence-based analysis of physical and chemical properties of reported (Sri) and novel transcript (Sri-N) using ExPaSy ProtParam tool.

Properties	Sri	Sri-N
Amino acid sequence	MAYPGHPGAGGGYYPGGYGGAPGGPAFPGQTQDPLYGYFAAVAGQDGQIDADELQRCLTQSGIAGGYKPFNLETCRLMVSMLDRDMSGTMGFNEFKELWAVLNGWRQHFISFDSDRSGTVDPQELQKALTTMGFRLSPQTVNSVAKRYSTSGKITFDDYIACCVKLRALTDSFRRRDSGQQGVVNFSYDD*FIQCVMTV*	MAYPGHPGAGGGYYPGGYGGAPGGPAFPGQTQDPLYGYFAAVAGQDGQIDADELQRCLTQSGIAGGYKPFNLETCRLMVSMLDRDMSGTMGFNEFKELWAVLNGWRQHFISFDSDRSGTVDPQELQKALTTMGFRLSPQTVNSVAKRYSTSGKITFDDYIACCVKLRALTDSFRRRDSGQQGVVNFSYDD*TLCDWSHFLSC*
Amino acids number	198	201
Molecular weight	21,627.26	21,998.57
Isoelectric Point (pI)	5.32	5.29
Net phos	S:13, Y:3, T:8	S:13, Y:4, T9
Predicted half-life (mammalian reticulocytes, in vitro)	30 h	30 h

**Table 2:** Sub cellular localization of Sri and Sri-N.

Cellular localization	Sri (%)	Sri-N (%)
Cytoplasm	60.9	60.9
Nuclear	13.0	13.0
Vesicle of secretory system	8.7	8.7
Endoplasmic reticulum	4.3	4.3
Golgi	4.3	4.3
Mitochondria	4.3	4.3

### Homology modelling and MD simulation analysis

**Figure 5:**
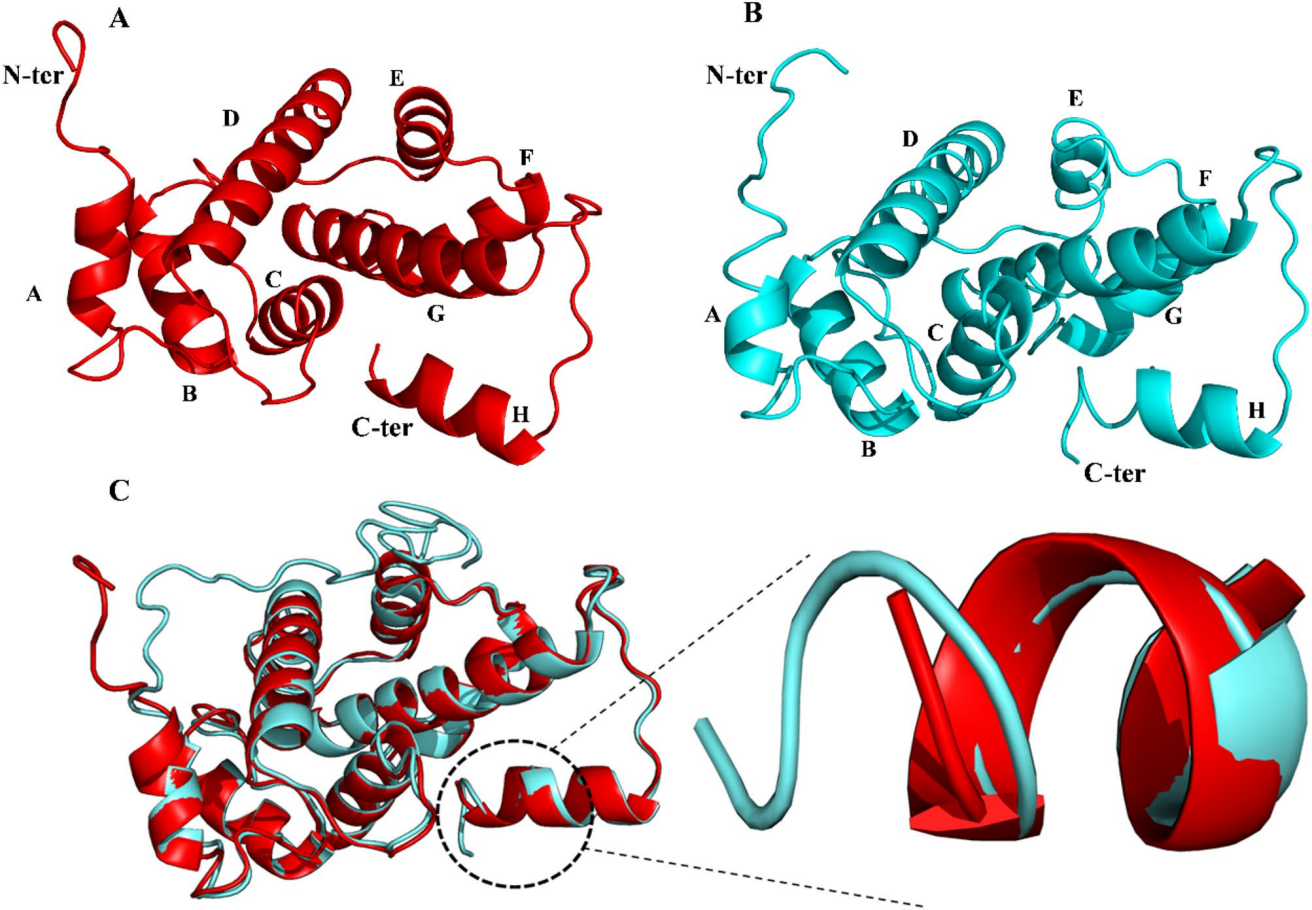
3D structure of Sri and the newly identified Sri-N. (**A**) 3D structure of Sri downloaded from RCBS PDB (PDB ID: 1juo) shown in red and the helices are labeled as A–H. (**B**) Homology modeling of the newly identified isoform Sri-N (cyan) using ITASSER server and the helices (A–H) are also indicated. (**C**) Overlapped structures of Sri (red) and the newly identified Sri-N (cyan) using I-TASSER and in this figure the blown up segment represents differences between structures of Sri and Sri-N at the C-terminal end. The visualization and alignment were done using PyMOL.

The protein with PDB ID: 1JUOA (crystal structure of calcium free human Sri) was taken as reference protein for Sri and was also utilized as template for homology modeling of the Sri-N protein using I-TASSER server. The pairwise sequence alignment generated an identity and similarity of 88.8% and 91.6% respectively for both isoforms. Both the isoforms were aligned using PyMOL and the RMSD of Sri-N with respect to Sri was calculated and was found to be 0.601 nm (Fig. 5C). The novel isoform was found to differ at the C-terminal end and the sequence alignment of Sri and Sri-N proteins showed the addition of 11 amino acids unique sequence in Sri-N that forms an extended α-helix (Fig. 5). The sequence wise secondary structure of Sri and Sri-N is given in the supplementary file (Fig. S1). Further analysis of their stability was examined by simulating both isoforms (novel and reported) by mimicking the physiological conditions for 100 ns at 310 K. The MD simulations of both the proteins were analyzed by measuring the RMSD, RMSF, *R*_g_, SASA, secondary structure prediction and hydrogen bond analysis.

**Figure 6:**
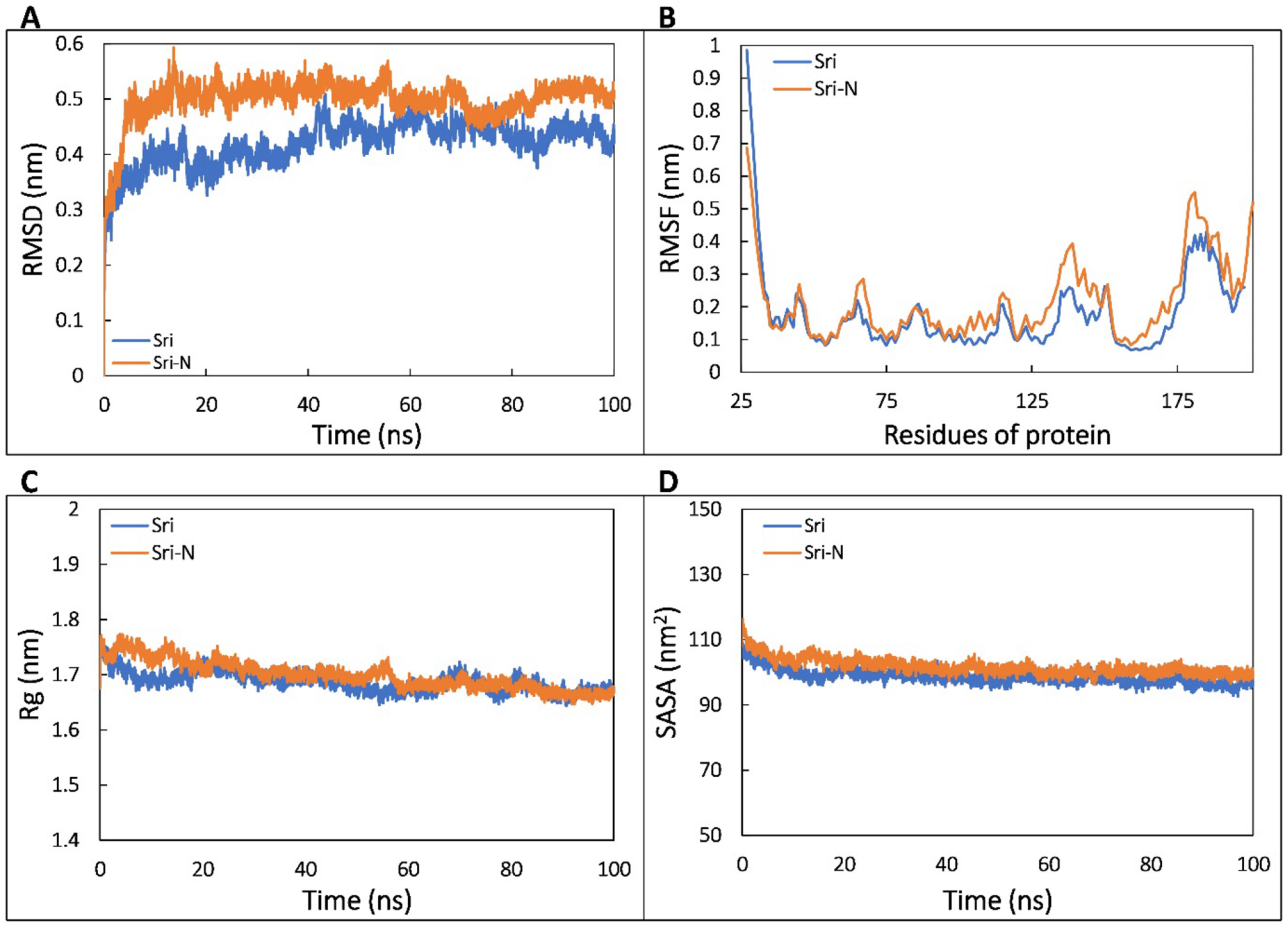
MD simulation analysis of Sri and Sri-N monomers. (**A**) Root mean square deviation (RMSD) of the backbone atoms of the reported (Sri) and the newly identified isoform (Sri-N) as a function of time. (**B**) Root mean square fluctuation (RMSF) of C_α_ atoms of Sri and Sri-N. (**C**) The radius of gyration (*R*_g_) of Sri and Sri-N as a function of time. (**D**) Solvent accessible surface area (SASA) of Sri and Sri-N as a function of time.

Root-mean square deviation (RMSD) is used to validate the stability of the protein structure by determining the deviations produced during MD simulation^21^. RMSD determines the structural difference in the backbones of a protein by comparing the deviation from its initial structural conformation to the positions of all frames of the trajectory. RMSD values of the backbone atoms of Sri and Sri-N isoforms are shown in Fig. 6A. The average RMSD values of Sri and Sri-N were 0.419 and 0.499 nm, respectively. As shown in Fig. 6A, it was observed that the system for reported (Sri) protein equilibrated after 20 ns and remained fairly stable throughout the trajectory. Approximately similar results were obtained for the novel isoform (Sri-N) where the system equilibrated at around 20 ns and remained stable after that. The average RMSD of Sri-N is slightly greater than Sri which is due to the large amount of irregular (coils, bends, and turns) structures. The sum of irregular structures in Sri and Sri-N is 44.01 and 51.72%, respectively.

The flexibility of the individual residues in a protein was calculated by determining the root-mean square fluctuation (RMSF). It calculates the time averaged position of a protein residue and depicts how much a particular amino acid fluctuates during the course of simulation^22^. The RMSF of C_α_ atoms of the novel protein displayed more flexible residues from 125 to 149 as compared to the reported protein. The region that showed higher RMSF values were mostly either coils, bends, or turns which tend to fluctuate in the aqueous environment. The average RMSF of Sri-N was also slightly higher than Sri which can be explained by the fact that Sri-N has more irregular structures compared to Sri (Fig. 6B). The compactness of the protein is measured by calculating the radius of gyration (*R*_g_) that tells the distribution of atoms of a protein around its axis during MD simulation. *R*_g_ values of the reported (Sri) and novel isoform (Sri-N) are represented in Fig. 6C. The average *R*_g_ of Sri was found to be 1.687 nm and that of the novel isoform was 1.699 nm suggesting stable nature of both the proteins. Furthermore, the stability of both the systems was verified by calculating the solvent accessible surface area (SASA). The average SASA of Sri-N was recorded as 101.708 nm^2^ as compared to reported Sri with the SASA value of 98.667 nm^2^ (Fig. 6D). The data represents negligible change in the SASA of both the protein models and consistency in the results confirms the stability of both the systems in physiological conditions. Furthermore, the secondary structure profile of both isoforms was investigated. The average percentage of coil, β-sheet, β-bridge, bend, turn, α-helix, and 3′-helix in Sri protein were found to be 15.93, 3.17, 0.84, 12.76, 15.30, 50.32, and 1.64, respectively. In contrast, the quantity of coil, β-sheet, β-bridge, bend, turn, α-helix, and 5′-helix, and 3′-helix in the Sri-N isoform was recorded as 18.00, 2.52, 2.15, 13.10, 20.61, 40.30, 0.15, and 3.13, respectively. Each of secondary structural components of Sri and Sri-N over the course of simulation is presented in the supplementary file (Fig. S2). The data infer that the novel isoform contains more coils, β-bridges, bends, turns, 5′-helices, and 3′-helices.

### Dimerization study using protein–protein docking and MD simulation

**Figure 7:**
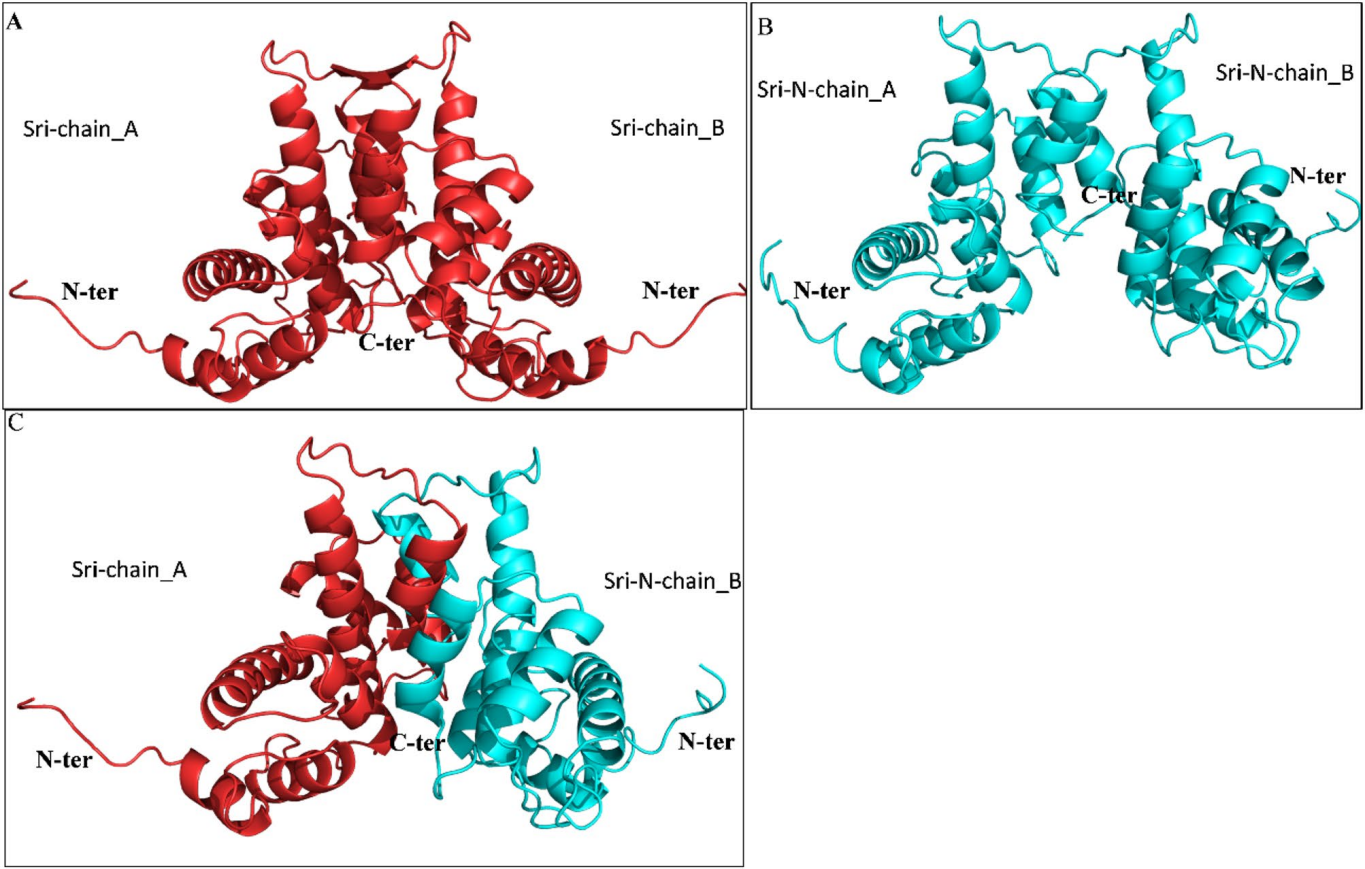
Structure validation of Sri and Sri-N dimers using ClusPro. (**A**) Interaction of Sri chain A and chain B to form Sri-Sri homodimer (red) using ClusPro server. (**B**) Interaction of Sri-N chain A and chain B to form Sri-N-Sri-N homodimer (cyan). (**C**) Interaction of Sri chain A (red) and Sri-N chain B (cyan) forming Sri-Sri-N heterodimer.

**Figure 8:**
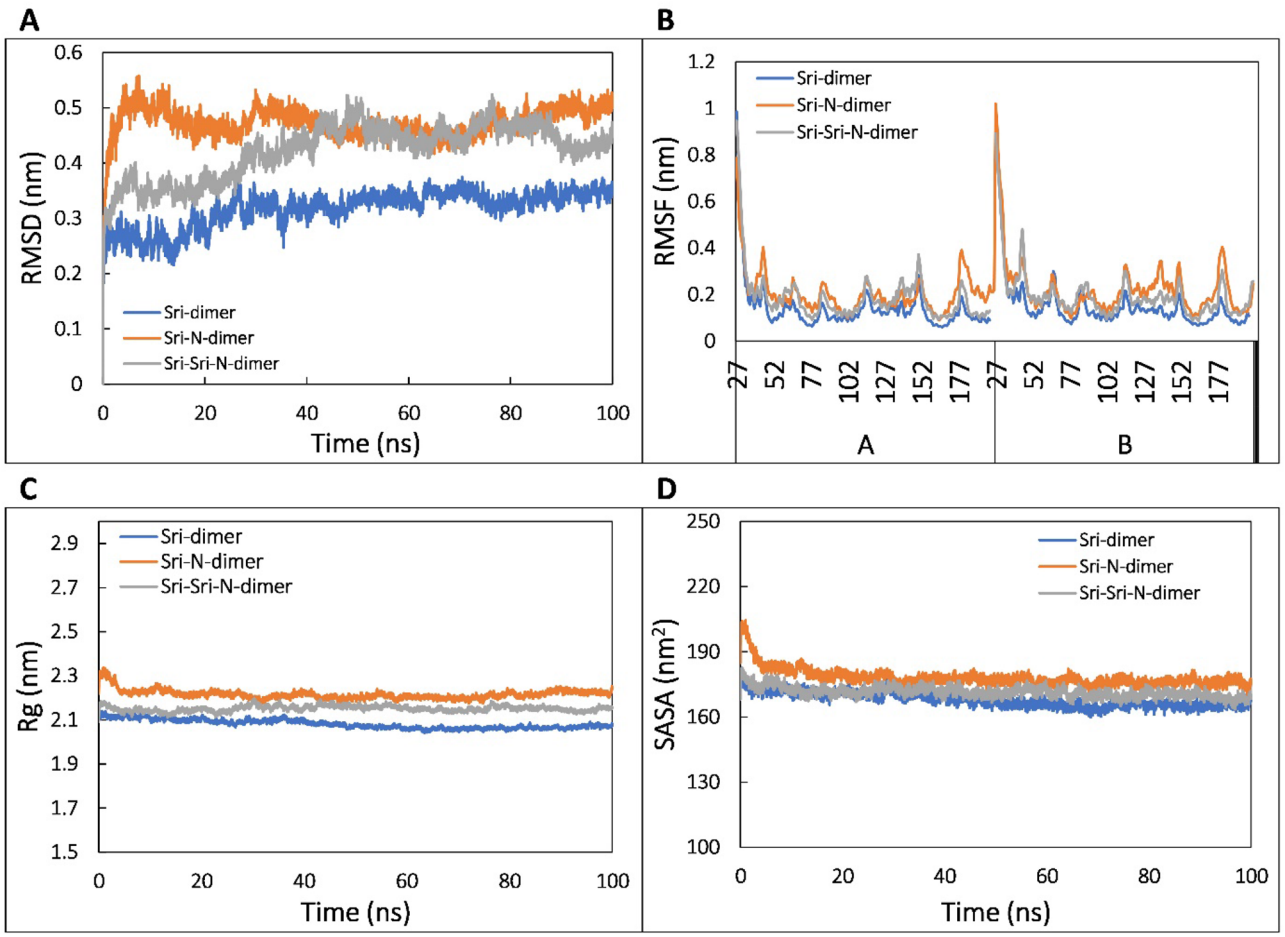
MD simulation analysis of dimers. (**A**) Root mean square deviation (RMSD) of backbone atoms of Sri-dimer (blue), Sri-N-dimer (red) and Sri-Sri-N (green) heterodimer as function of time. (**B**) Root mean square fluctuation (RMSF) of C_α_ atoms of Sri-dimer (blue), Sri-N-dimer (red) and Sri-Sri-N (green) heterodimer. (**C**) Radius of gyration (*R*_g_) of Sri-dimer (blue), Sri-N-dimer (red) and Sri-Sri-N (green) heterodimer as function of time. (**D**) Solvent accessible surface area (SASA) of Sri-dimer (blue), Sri-N-dimer (red) and Sri-Sri-N (green) heterodimer as function of time.

The crystal structure of human Sri displays its globular shape that forms homodimer structure. Therefore, the monomeric protein structure obtained by ITASSER was used to check the protein–protein interaction. We studied 3 different dimers, namely, [Sri]–[Sri] homodimer, [Sri-N]–[Sri-N] homodimer and [Sri]–[Sri-N] heterodimer (as shown in Fig. 7, Supplementary Fig. S3) and proceeded with the structures having lowest binding energy for MD simulation to check the stability of the dimers. The average RMSD values for [Sri]–[Sri] homodimer, [Sri-N]–[Sri-N] homodimer and [Sri]–[Sri-N] heterodimer was recorded as 0.316, 0.473, 0.422 nm, respectively. From the RMSD simulation graph, shown in Fig. 8A, it can be seen that [Sri]–[Sri] homodimer system equilibrated after 30 ns and remained stable throughout the simulation, whereas for [Sri-N]–[Sri-N] homodimer, the equilibration was obtained within 10 ns and became stable. A similar result was obtained for heterodimer [Sri]–[Sri-N] in which the system became stable after 40 ns of the trajectory. The average RMSF values of C_α_ atoms of [Sri]–[Sri], [Sri-N]–[Sri-N] and [Sri]–[Sri-N] were calculated as 0.116, 0.108, and 0.109 nm, respectively (Fig. 8B).

To study the compactness of the dimers, the radius of gyration (*R*_g_) was calculated for each dimer and average *R*_g_ values of 2.081 and 2.216 nm was obtained for [Sri]–[Sri] homodimer, [Sri-N]–[Sri-N] homodimer, respectively. The average *R*_g_ for [Sri]–[Sri-N] heterodimer was found to be 2.151 nm. The graph shown in Fig. 8C represents the comparison of *R*_g_ values between all the 3 dimers over the course of simulation. The variations within the trajectories are acceptable considering the flexibility of proteins in an aqueous environment.

The structural compactness and stability of protein dimers were also examined by calculating the change in SASA of all systems. The average SASA values of [Sri]-[Sri] homodimer, [Sri-N]–[Sri-N] homodimer and [Sri]–[Sri-N] heterodimer was recorded as 167.964, 178.149, and 171.527 nm^2^, respectively (Fig. 8D).

### Protein ligand docking analysis

The molecular docking study was performed to compare the intermolecular interaction of different chemotherapeutic drugs with Sri and Sri-N isoforms. It is an extremely convenient method to determine the top binding conformation of a ligand molecule to their receptor. Earlier studies have revealed the high affinity of different chemotherapeutic drugs with Sri at the SCBD domain^23^. In the current study, we have selected 4 chemotherapeutic drugs, namely doxorubicin, etoposide, vincristine and omacetaxine that bind to Sri at the C-terminal domain^5,18,19^, and they were docked with both Sri and Sri-N using AutoDock Vina so as to compare the difference in their binding energies. The lowest binding energies of all the drugs with Sri and Sri-N are listed in Table 3. Previous investigations through SPR and fluorescence titration experiments have validated the presence of two doxorubicin binding-sites (pocket 1 and 2) where pocket 1 has reported to have the highest *K*_d_ value of 1.4 ± 1 nM^18^. Furthermore, the crystallization studies have obtained the 3D structure of Sri-doxorubicin complex at this site (pocket-1), that shows the binding site at the interface between the EF5 loop and G-helix of EF4 loop.

**Table 3:** Binding energy of different chemotherapeutic drugs with Sri isoforms.

Drug	Sri (kcal/mol)	Sri-N (kcal/mol)
Doxorubicin	− 7.3	− 8.3
Etoposide	− 8.1	− 8.1
Vincristine	− 7.4	− 7.9
Omacetaxine	− 6.5	− 6.9

**Figure 9:**
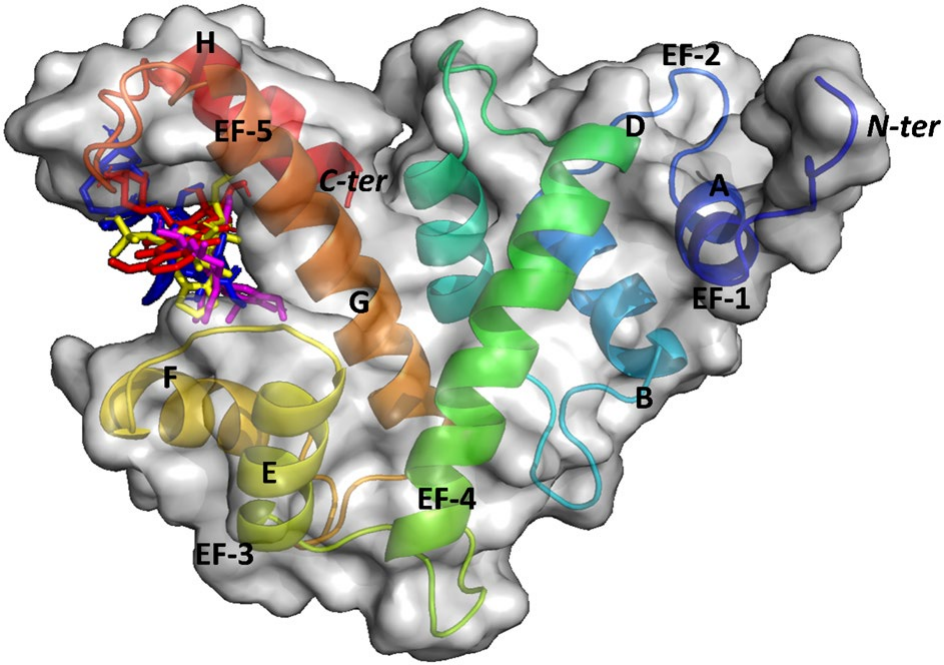
Docked conformations of different ligands with Sri at the interface between EF5 loop and G-helix. Conformation of different compounds is shown by colored sticks and that of Sri by rainbow colored cartoon surfaced in grey: Red stick, doxorubicin; magenta stick, etoposide; yellow stick, omacetaxine; blue stick, vincristine.

**Figure 10:**
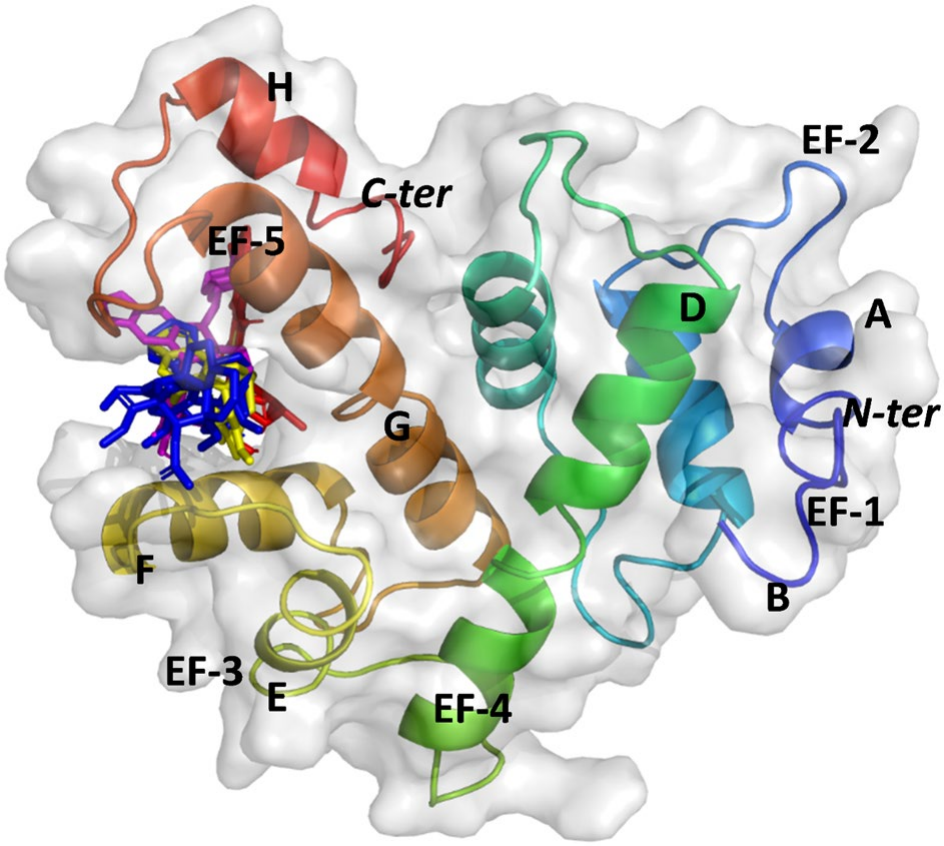
Docked conformations of different ligands with Sri-N. Molecular model of Sri-N complexed with doxorubicin, etoposide, omacetaxine and vincristine shown by red, magenta, yellow and blue colors, respectively at the interface between the EF5 loop and the G-helix.

Consequently, we compared the intermolecular interaction and binding energies of all four drugs with Sri and Sri-N and ascertained that all the chemotherapeutic drugs chosen were able to bind the active pocket, i.e., pocket 1 present at the interface between EF5 loop and the G-helix of both Sri and Sri-N as shown in Figs. 9 and 10. The binding energy of doxorubicin, etoposide, vincristine and omacetaxine with Sri was calculated as − 7.3, − 8.1, − 7.4 and − 6.5 kcal/mol, respectively. In contrast, values of binding energy of doxorubicin, etoposide, vincristine and omacetaxine with Sri-N were − 8.3, − 8.1, − 7.9 and − 6.9 kcal/mol, respectively. These observations clearly show that the binding affinity of doxorubicin, vincristine and omacetaxine was more for Sri-N as compared to that for the reported protein (Sri).

The most common residues within the pocket of Sri to which all the drugs forms hydrogen bond include Thr170 and Val195 whereas in case of Sri-N, Leu169, Leu166, Thr170 and Gly-182 were involved in the hydrogen bonding. Moreover, etoposide, omacetaxine and vincristine were able to form hydrogen bond with Leu166 of both Sri and Sri-N (Supplementary Figs. S4–S7). To study the dynamics and stability of complexes, we have also performed short simulation of all complexes. The RMSD, RMSF, Rg, and SASA of all systems were found to be stable (Supplementary Figs. S8, S9). Moreover, MM-PBSA binding energies were also calculated which is more accurate than the binding energies obtained from molecular docking (Supplementary Fig. S10). The overall energy for complexation of doxorubicin, etoposide, omacetaxine, and vincristine to Sri was found to be − 28.79, − 28.82, − 36.63, and − 43.67 kcal/mol, respectively. Similarly, overall energy for complexation of doxorubicin, etoposide, omacetaxine, and vincristine to Sri-N was − 41.34, − 28.50, − 35.90, and − 37.92 kcal/mol, respectively.

## Discussion

Sri is a soluble, calcium binding protein and widely expressed in various tissues. It is also reported to be overexpressed in many human cancers such as leukemias, lymphomas, breast, nasopharyngeal, lung, adenocarcinoma and has a significant role in multidrug resistance (MDR). Sri belongs to the members of penta-EF-hand (PEF) protein family that contains helix-loop-helix motifs that are able to bind calcium with high affinity and regulate a variety of cellular processes. Sri is encoded by SRI gene located on chromosome number 7 and 5 in humans and mouse, respectively. In humans, SRI gene generates 4 different isoforms as a result of alternative splicing whereas 2 isoforms have been reported in the mouse as per Mouse Genome Informatics (MGI) database. Alternative splicing is a process that plays a significant role in providing complexity in terms of transcriptomics and proteomics in multi-cellular organisms. It can generate multiple transcripts from a single gene with different structures and functions^24^. It has been increasingly recognized in many forms of diseases such as cancer, apoptosis, metabolic disorders and other physiological pathways^25^.

In this study, we have identified a novel, alternatively spliced transcript variant of mouse Sri gene using a combination of bioinformatics and molecular biology techniques. The previously reported isoforms differ at their N-terminal region whereas the novel isoform, Sri-N, identified in this study has a different C-terminal region. A novel exon with a stop codon (E8ʹ) was identified to be located in the 3ʹ UTR using FEX tool. This exon participates in alternative splicing with the second last coding exon (E7), replacing the last coding exon (E8) from the mature transcript. This leads to the generation of a distinct C-terminal caused by the replacement of 8 amino acid sequence with 11 novel amino acid sequence in Sri-N. The expression of novel isoform was confirmed, at transcript level, in the pancreas, adipose and liver tissues. Sri-N also showed similar subcellular localization pattern as studied using PSORT-II server (Table 2). The structural stability and variations of Sri isoforms were also studied using MD simulation and the RMSD, RMSF, *R*_g_, SASA, potential energy, total energy and secondary structure plots were compared. The overall deviation in the trajectory of the RMSD plot, similar fluctuation in the RMSF, and an approximately similar *R*_g_ values suggested the stability and a compact nature of Sri-N.

The X-ray crystal structure of Sri has deciphered that the protein is globular in shape and forms homodimer^26^. The dimer formation capabilities of Sri are majorly due to the amino acids located towards the C-terminal side. Since Sri and Sri-N isoforms differs in the C-terminal tail, protein–protein interaction study was performed, and stability of the dimers were studied using MD simulation. It was observed that Sri-N forms more stable homodimer compared to Sri. However, Sri-Sri-N heterodimer was relatively less stable. The findings suggested that the dimer formation capabilities of novel Sri-N were intact and may not lead to any loss of function.

Sri was first isolated as a soluble protein from the hamster lung tumor cells that were found to be resistant to vincristine^27^. It was found to be co-amplified with ABC1 gene in MD-resistant cancer cells, and therefore it was labelled as soluble resistant related calcium binding protein. In addition to its wide expression, Sri is overexpressed in many human tumors where its overexpression confers increased resistance to many chemotherapeutic drugs leading to MDR in human cancer cell lines^28^. Sri takes part in various processes such as drug efflux regulation, apoptosis modulation and epithelial-to-mesenchymal transition (EMT) control that aids in multidrug resistance (MDR) in human cancers^3^. It is also reported that when the expression of Sri is decreased, cells become more sensitive to doxorubicin which is a chemotherapeutic drug^29^. Also, Sri expression is reported to be associated with the upregulation of MDR1 in leukemia cells^30^. Interaction between several chemotherapeutic drugs and Sri has been reported. As per earlier reports, the selected chemotherapeutics drugs were found to bind at the interface among the EF5 loop, G helix EF4 loop of pocket1^19^. To check the intermolecular interaction of Sri-N with chemotherapeutic drugs (doxorubicin, etoposide, omacetaxine and vincristine), molecular docking studies was performed between Sri-N and different drugs. Interestingly, Doxorubicin, Vincristin and Omacetaxine were found to interact with Sri-N with slightly higher affinity compared to Sri. These observations require further validation that will ascertain the role of novel Sri isoform in multi drug resistance (MDR). It would also be interesting to study the expression level of Sri-N in different tumor cells.

### Supplementary Information

Supplementary Figures.

## Data Availability

All data generated or analyzed during this study are included in this article.

## References

[1] Zhou X, Wu X, Chen B (2019). Sorcin: A novel potential target in therapies of cancers. Cancer Manag. Res..

[2] Wang Y, Zhu Y, Pu Z, Li Z, Deng Y, Li N, Peng F (2021). Soluble resistance-related calcium-binding protein participates in multiple diseases via protein–protein interactions. Biochimie.

[3] Colotti G, Poser E, Fiorillo A, Genovese I, Chiarini V, Ilari A (2014). Sorcin, a calcium binding protein involved in the multidrug resistance mechanisms in cancer cells. Molecules.

[4] Ilari A, Johnson KA, Nastopoulos V, Verzili D, Zamparelli C, Colotti G, Tsernoglou D, Chiancone E (2002). The crystal structure of the Sorcin calcium binding domain provides a model of Ca2+-dependent processes in the full-length protein. J. Mol. Biol..

[5] Battista T, Fiorillo A, Chiarini V, Genovese I, Ilari A, Colotti G (2020). Roles of Sorcin in drug resistance in cancer: One protein, many mechanisms, for a novel potential anticancer drug target. Cancers.

[6] Lalioti VS, Ilari A, O’Connell DJ, Poser E, Sandoval IV, Colotti G (2014). Sorcin links calcium signaling to vesicle trafficking, regulates Polo-like kinase 1 and is necessary for mitosis. PLoS ONE.

[7] Ilari A, Fiorillo A, Poser E, Lalioti VS, Sundell GN, Ivarsson Y, Genovese I, Colotti G (2015). Structural basis of Sorcin-mediated calcium-dependent signal transduction. Sci. Rep..

[8] Zamparelli C, Ilari A, Verzili D, Giangiacomo L, Colotti G, Pascarella S, Chiancone E (2000). Structure–function relationships in Sorcin, a member of the penta EF-hand family. Interaction of Sorcin fragments with the ryanodine receptor and an *Escherichia coli* model system. Biochemistry.

[9] Kornblihtt AR, Schor IE, Alló M, Dujardin G, Petrillo E, Muñoz MJ (2013). Alternative splicing: A pivotal step between eukaryotic transcription and translation. Nat. Rev. Mol. Cell Biol..

[10] Genovese I, Ilari A, Battista T, Chiarini V, Fazi F, Fiorillo A, Colotti G (2018). Molecular bases of Sorcin-dependent resistance to chemotherapeutic agents. Cancer Drug Resist..

[11] Zhou Y, Xu Y, Tan Y, Qi J, Xiao Y, Yang C, Zhu Z, Xiong D (2006). Sorcin, an important gene associated with multidrug-resistance in human leukemia cells. Leuk. Res..

[12] Ishqi HM, Husain MA, Rehman SU, Sarwar T, Tabish M (2018). Identification and expression of alternatively spliced novel isoforms of cancer associated MYD88 lacking death domain in mouse. Mol. Biol. Rep..

[13] Ishqi HM, Ur Rehman S, Sarwar T, Husain MA, Tabish M (2016). Identification of differentially expressed three novel transcript variants of mouse ARNT gene. IUBMB Life.

[14] Nakai K, Horton P (1999). PSORT: A program for detecting sorting signals in proteins and predicting their subcellular localization. Trends Biochem. Sci..

[15] Van Der Spoel D, Lindahl E, Hess B, Groenhof G, Mark AE, Berendsen HJC (2005). GROMACS: Fast, flexible, and free. J. Comput. Chem..

[16] Hornak V, Abel R, Okur A, Strockbine B, Roitberg A, Simmerling C (2006). Comparison of multiple Amber force fields and development of improved protein backbone parameters. Proteins Struct. Funct. Bioinform..

[17] Desta IT, Porter KA, Xia B, Kozakov D, Vajda S (2020). Performance and its limits in rigid body protein–protein docking. Structure.

[18] Genovese I, Fiorillo A, Ilari A, Masciarelli S, Fazi F, Colotti G (2017). Binding of doxorubicin to SorcinSri impairs cell death and increases drug resistance in cancer cells. Cell Death Dis..

[19] Altharawi A, Ahmad S, Alamri MA, ul Qamar MT (2021). Structural insight into the binding pattern and interaction mechanism of chemotherapeutic agents with Sorcin by docking and molecular dynamic simulation. Colloids Surf. B Biointerfaces.

[20] Trott O, Olson AJ (2009). AutoDock Vina: Improving the speed and accuracy of docking with a new scoring function, efficient optimization, and multithreading. J. Comput. Chem..

[21] Fouedjou RT, Chtita S, Bakhouch M, Belaidi S, Ouassaf M, Djoumbissie LA, Tapondjou LA, Abul QF (2022). Cameroonian medicinal plants as potential candidates of SARS-CoV-2 inhibitors. J. Biomol. Struct. Dyn..

[22] Berman HM, Westbrook J, Feng Z, Gilliland G, Bhat TN, Weissig H, Shindyalov IN, Bourne PE (2000). The protein data bank. Nucleic Acids Res..

[23] Graveley BR (2001). Alternative splicing: Increasing diversity in the proteomic world. Trends Genet..

[24] Kim HK, Pham MH, Ko KS, Rhee BD, Han J (2018). Alternative splicing isoforms in health and disease. Pflügers Archiv.-Eur. J. Physiol..

[25] Xie X, Dwyer MD, Swenson L, Parker MH, Botfield MC (2001). Crystal structure of calcium-free human sorcin: A member of the penta-EF-hand protein family. Protein Sci..

[26] Meyers MB, Biedler JL (1981). Increased synthesis of a low molecular weight protein in vincristine-resistant cells. Biochem. Biophys. Res. Commun..

[27] Shabnam B, Padmavathi G, Banik K, Girisa S, Monisha J, Sethi G, Fan L, Wang L, Mao X, Kunnumakkara AB (2018). Sorcin a potential molecular target for cancer therapy. Transl. Oncol..

[28] Aier I, Varadwaj PK, Raj U (2016). Structural insights into conformational stability of both wild-type and mutant EZH2 receptor. Sci. Rep..

[29] Genovese I, Fiorillo A, Ilari A, Masciarelli S, Fazi F, Colotti G (2017). Binding of doxorubicin to Sorcin impairs cell death and increases drug resistance in cancer cells. Cell Death Dis..

[30] Yamagishi N, Nakao R, Kondo R, Nishitsuji M, Saito Y, Kuga T (2014). Increased expression of sorcin is associated with multidrug resistance in leukemia cells via up-regulation of MDR1 expression through cAMP response element-binding protein. Biochem. Biophys. Res. Commun..

