# Supplementary material for: Identification of a novel Sorcin isoform with a different C-terminal but intact dimerization property
Source: Sci Rep. 2023 Sep 14;13:15262. doi: 10.1038/s41598-023-40913-z (PMC10502117; doi:10.1038/s41598-023-40913-z)
Supplement: Supplementary file 1 — Supplementary Figures. [file 41598_2023_40913_MOESM1_ESM.docx]

**Identification of a novel Sorcin isoform with a different C-terminal but intact dimerization property**

**Supriya Tanwar^1^, Faizan Abul Qais^2^, Farheen Naaz^1^, Naira Rashid^1^, Faizan Ahmad^1^, and Sayeed ur Rehman^1^***

**^1^**Department of Biochemistry, School of Chemical and Life Sciences, Jamia Hamdard, New Delhi-110062, India

**^2^**Department of Agricultural Microbiology, Faculty of Agricultural Sciences, Aligarh Muslim University, Aligarh, UP 202002, India

**SUPPLEMENTARY FIGURES**


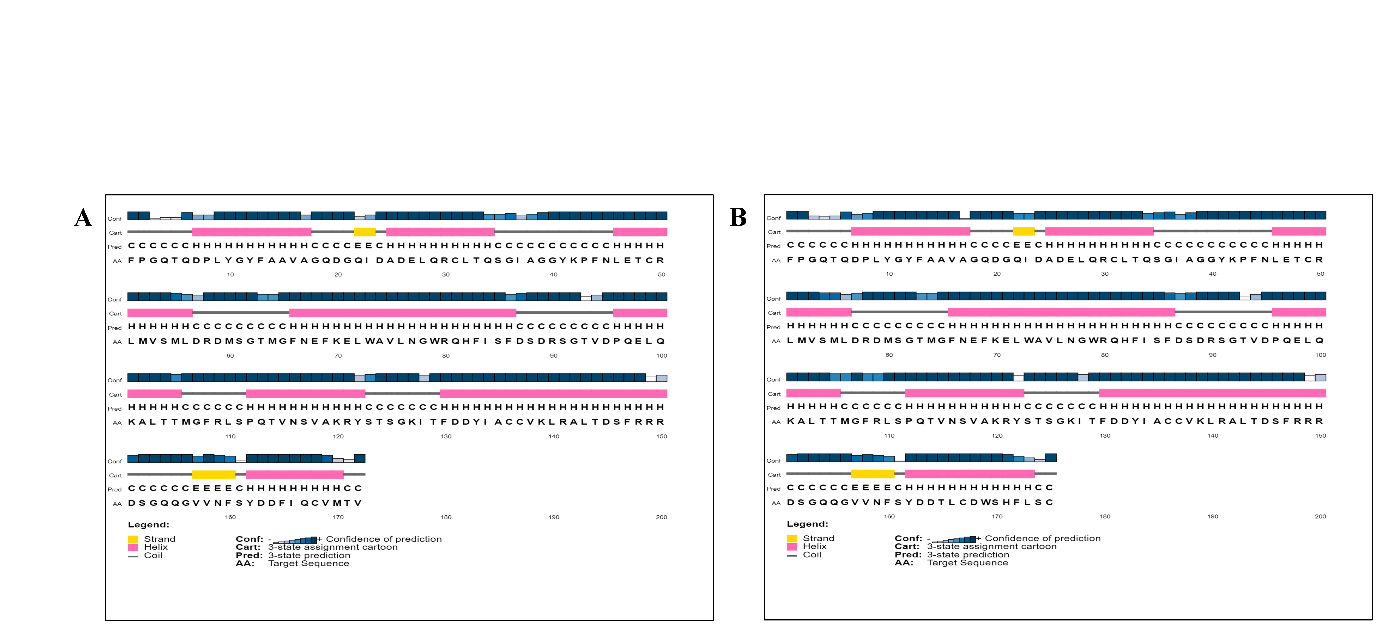


**Supplementary Fig S1**. **(A)** The graphical representation of secondary structure of the Sri predicted by PSIPRED. **(B)** Secondary structure prediction of Sri-N by PSIPRED.


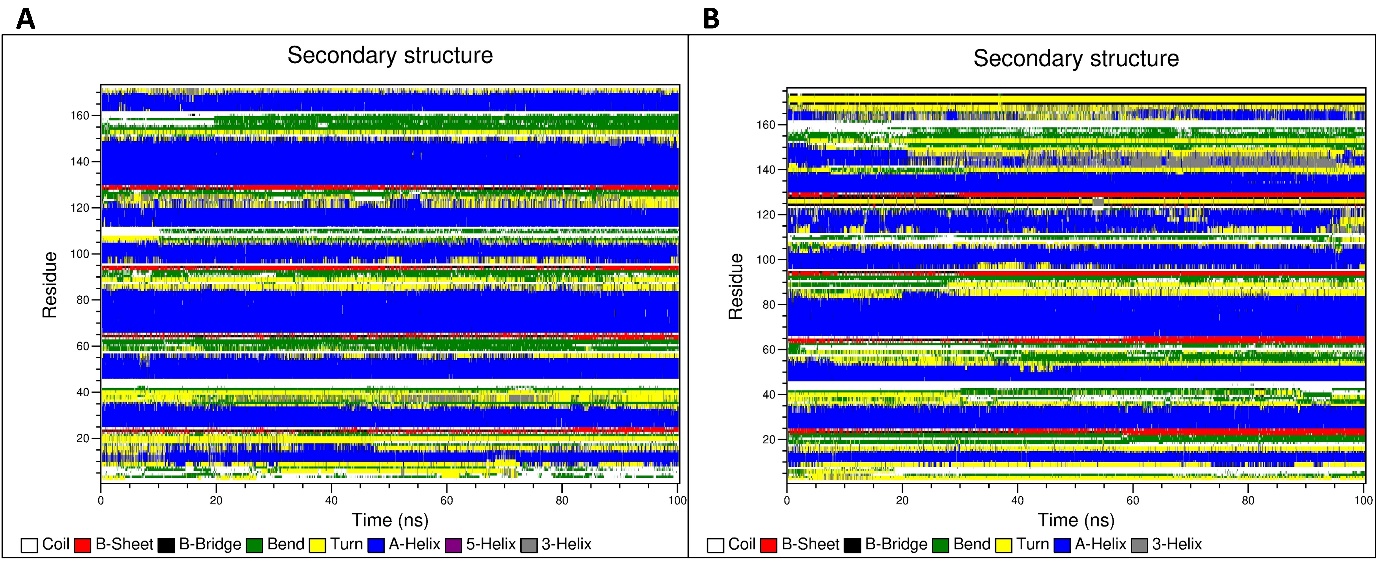


**Supplementary Fig S2.** **(A)** Each of the secondary structural components of Sri during the course of simulation. **(B)** Each of the secondary structural components of Sri-N during the course of simulation.


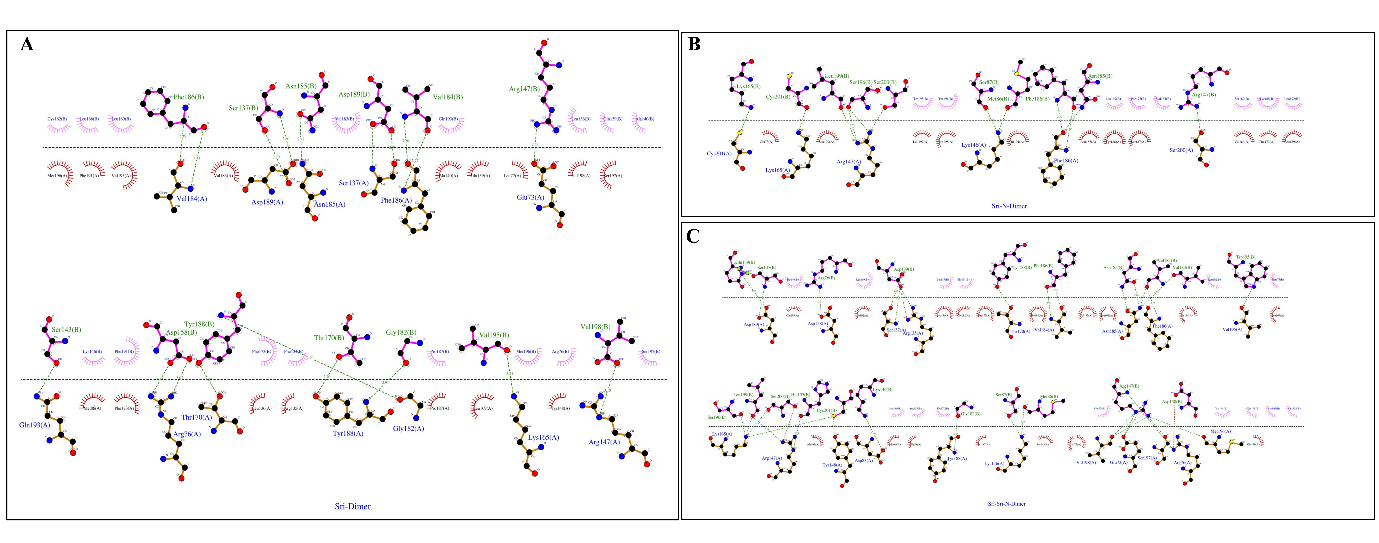


**Supplementary Fig S3. (A)** 2-D representation of interactions between Sri-Sri homodimer using LigPlot+. **(B)** 2-D scheme of interactions between Sri-N-Sri-N homodimer using LigPlot+. **(C)** 2-DRepresentation of interactions between Sri-Sri-N heterodimer using LigPlot+. (Hydrogen bonds are coloured in green dashed lines).


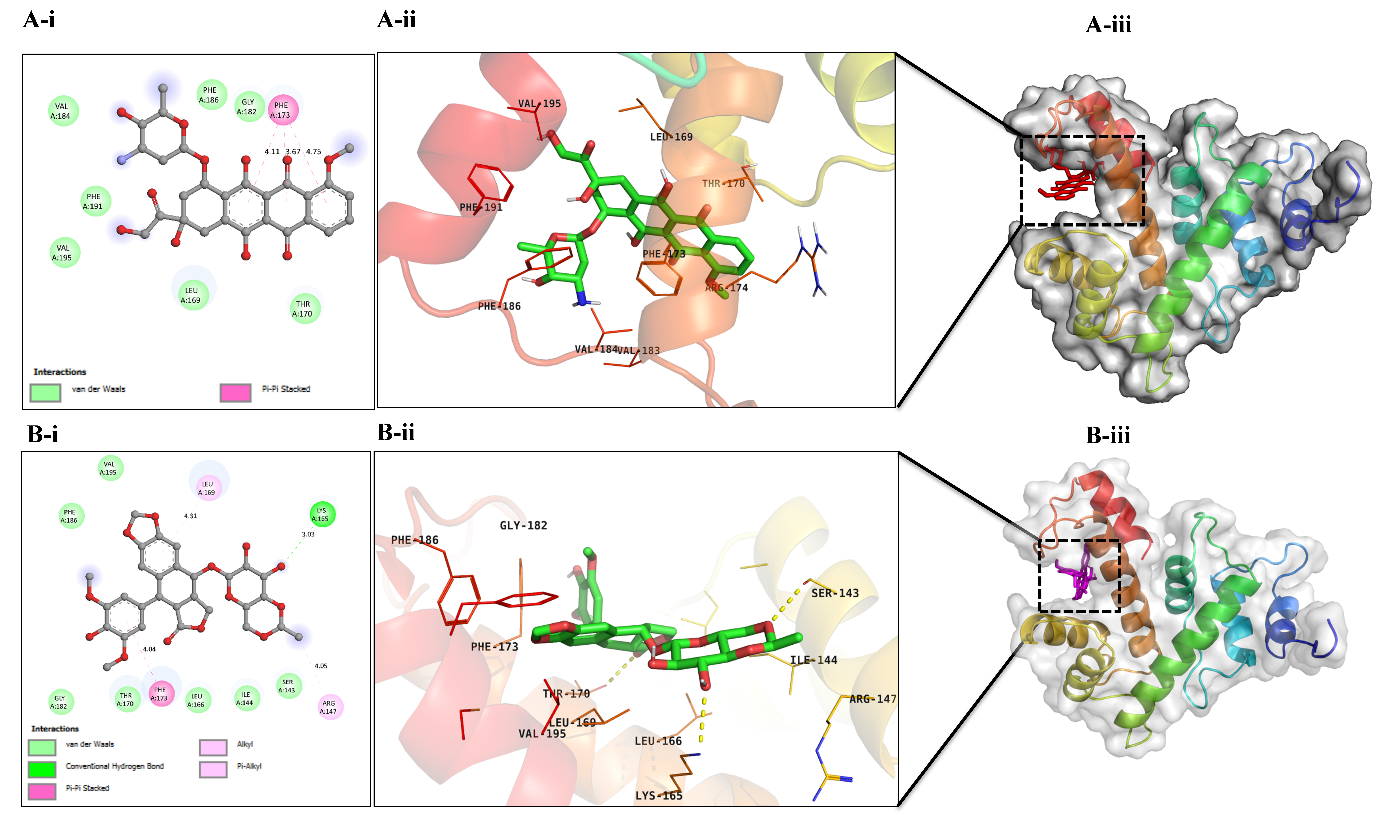


**Supplementary Fig S4. (A)** (i) 2D representation of Sri along with doxorubicin by Discovery studio showing hydrogen bonds in dash green lines and corresponding bond distance (ii) 3-D representation of Sri with doxorubicin by PyMOL (iii) Detailed view of the docking of doxorubicin (red colour stick) with Sri. (**B)** (i) 2D representation of Sri along with Etoposide by Discovery studio showing hydrogen bonds in dash green lines and corresponding bond distance (ii) 3-D representation of Sri with Etoposide by PyMOL (iii) Detailed view of the docking of Etoposide (magenta colour stick) with Sri.


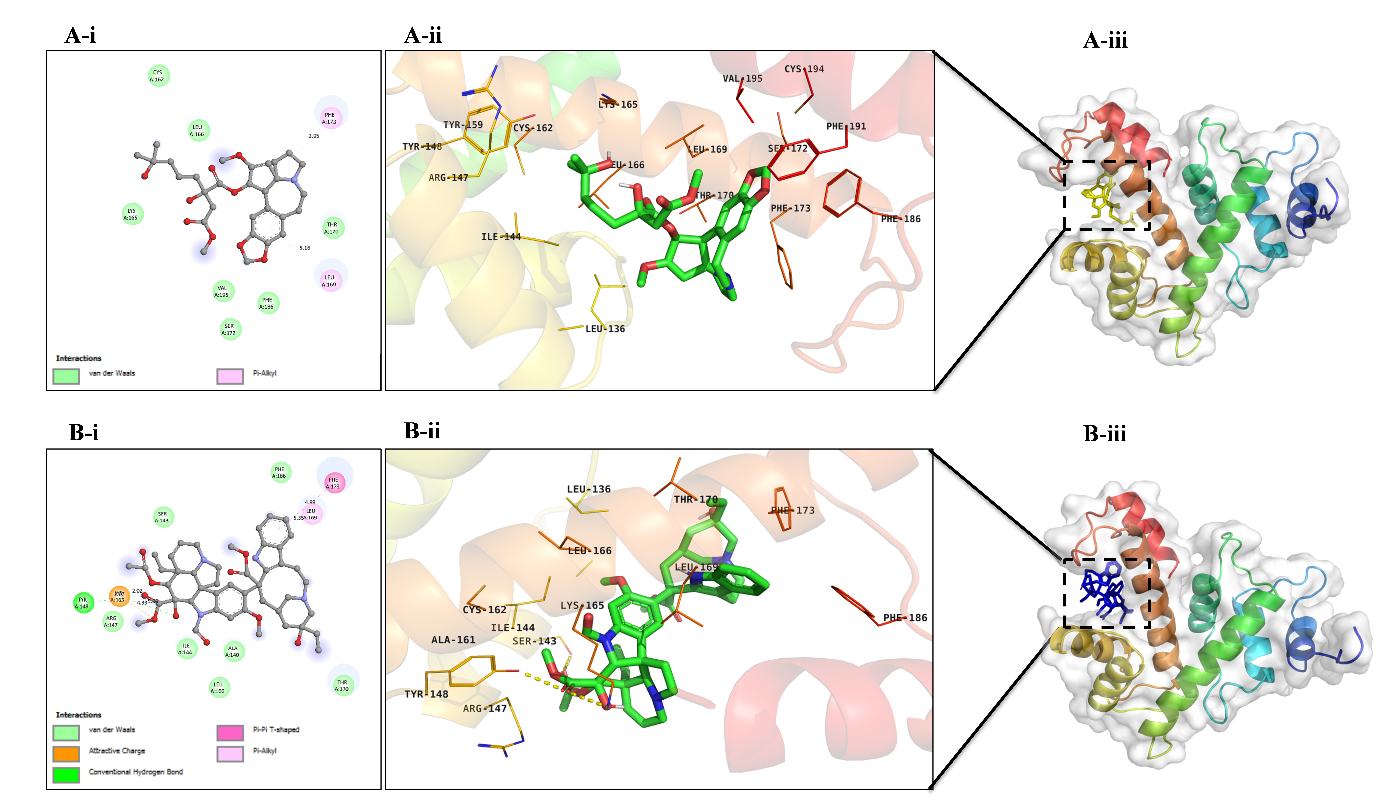


**Supplementary Fig S5. (A)** (i) 2D representation of Sri with omacetaxine by Discovery studio displaying hydrogen bonds (dash green lines) and their bond distance (ii) 3D representation of Sri with omacetaxine using PyMOL (iii) Detailed view of docking of omacetaxine (yellow colour stick) with Sri. (**B)** (i) 2D structure showing comprehensive docking details of vincristine with Sri by Discovery studio indicating hydrogen bonds in dash green lines and the respective bond distance (ii) 3D representation of Sri with vincristine using PyMOL. (iii) Detailed view of docking of vincristine (blue colour stick) with Sri.


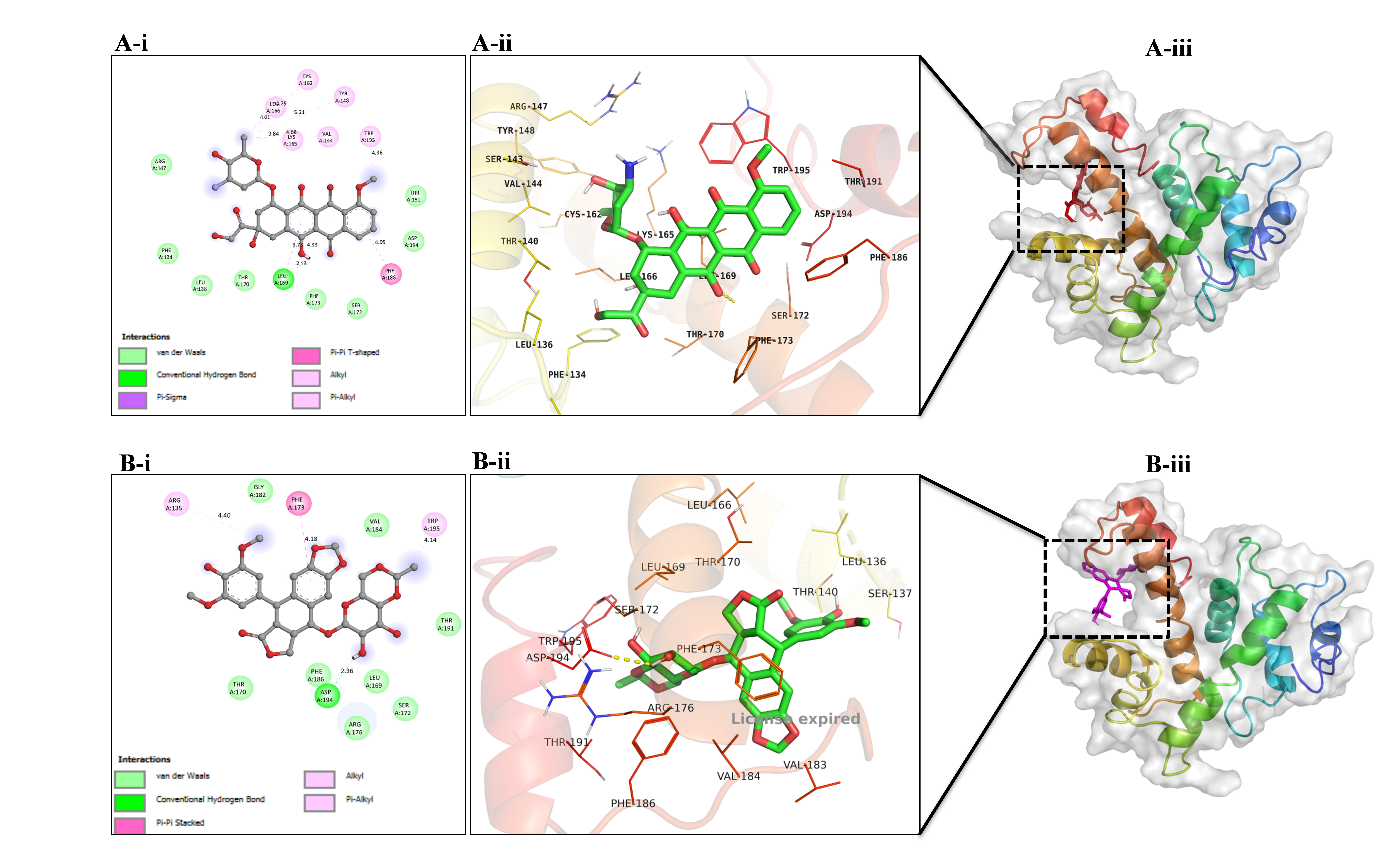


**Supplementary Fig S6. (A)** (i) 2D representation of Sri-N with doxorubicin by Discovery studio displaying hydrogen bonds (dash green lines) and their bond distance (ii) 3D representation of Sri-N with doxorubicin using PyMOL (iii) Detailed view of docking of doxorubicin (red colour stick) with Sri-N. (**B)** (i) 2D structure showing comprehensive docking details of etoposide with Sri-N by Discovery studio indicating hydrogen bonds in dash green lines and the respective bond distance (ii) 3D representation of Sri-N with etoposide using PyMOL. (iii) Detailed view of docking of etoposide (magenta colour stick) with Sri-N.


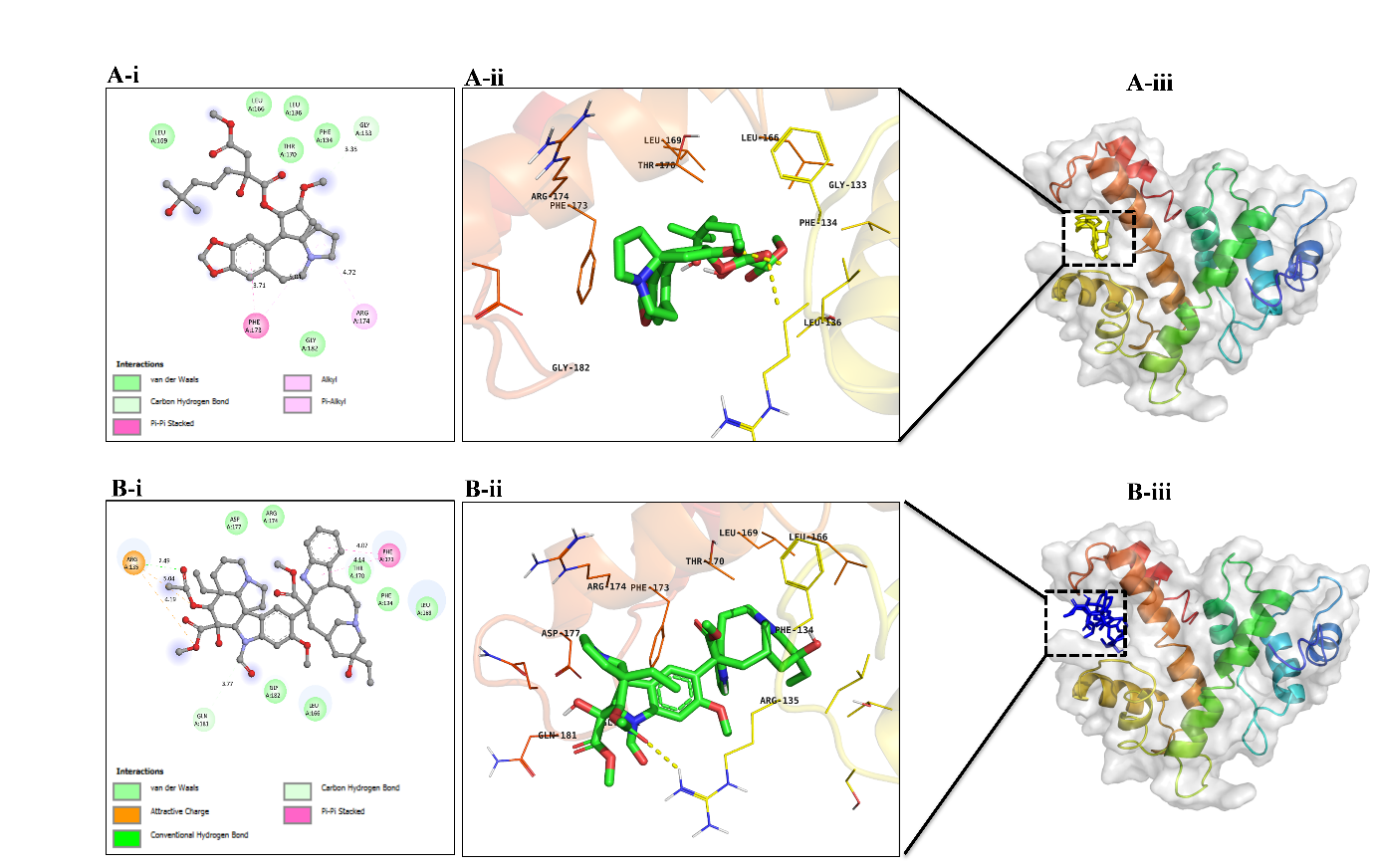


**Supplementary Fig S7. (A)** (i) 2D representation of Sri-N with omacetaxine by Discovery studio displaying hydrogen bonds (dash green lines) and their bond distance (ii) 3D representation of Sri-N with omacetaxine using PyMOL (iii) Detailed view of docking of omacetaxine (yellow colour stick) with Sri-N. (**B)** (i) 2D structure showing comprehensive docking details of vincristine with Sri-N by Discovery studio indicating hydrogen bonds in dash green lines and the respective bond distance (ii) 3D representation of Sri-N with vincristine using PyMOL. (iii) Detailed view of docking of vincristine (blue colour stick) with Sri-N.


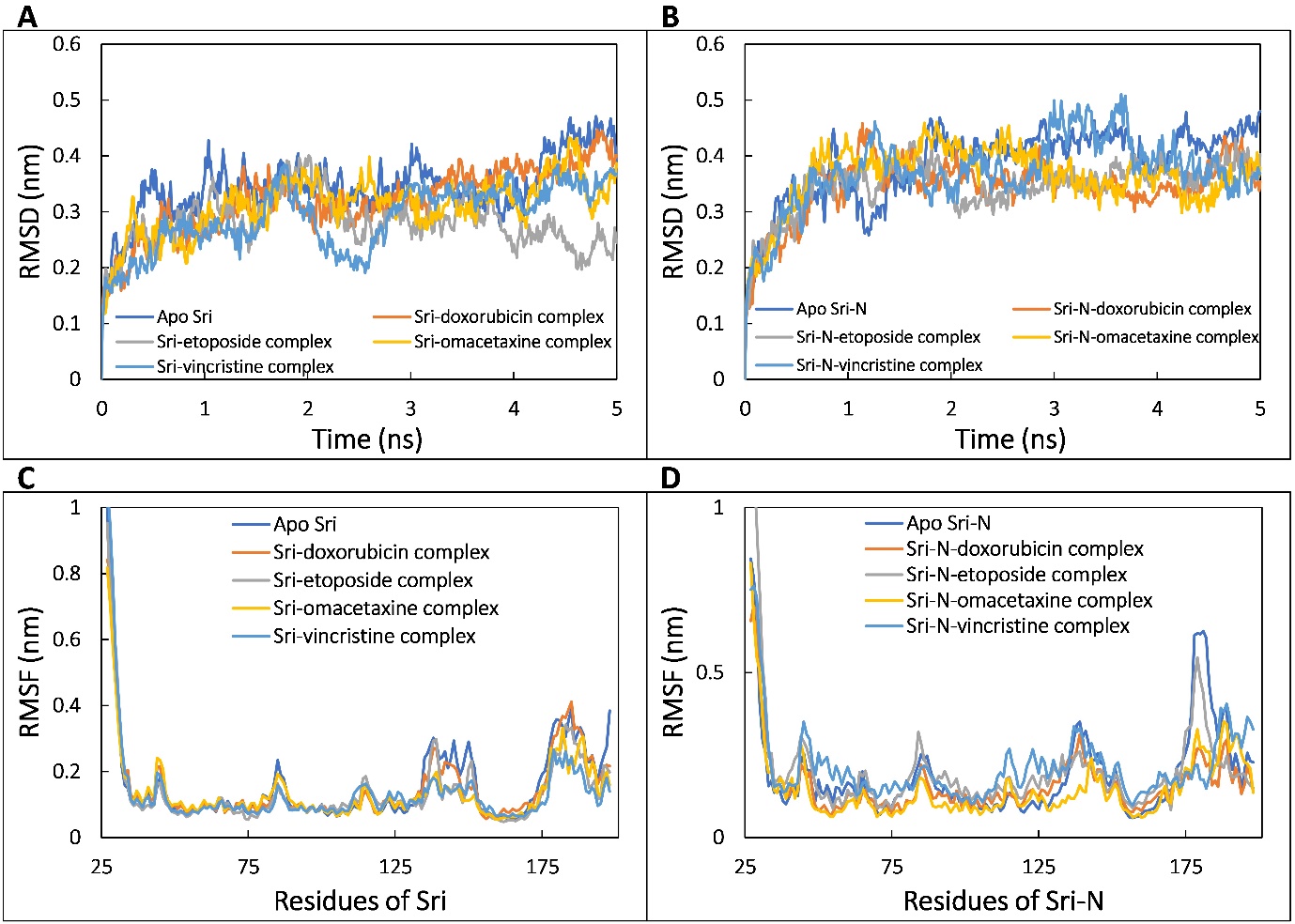


**Supplementary Fig S8.** **(A)** Root mean square deviation (RMSD) of the backbone atoms of the reported (Sri) in the absence and presence of ligands. **(B)** Root mean square deviation (RMSD) of the backbone atoms of the novel (Sri-N) in the absence and presence of ligands. **(C)** Root mean square fluctuation (RMSF) of Cα atoms of Sri in the absence and presence of ligands. **(D)** Root mean square fluctuation (RMSF) of Cα atoms of Sri-N in the absence and presence of ligands.


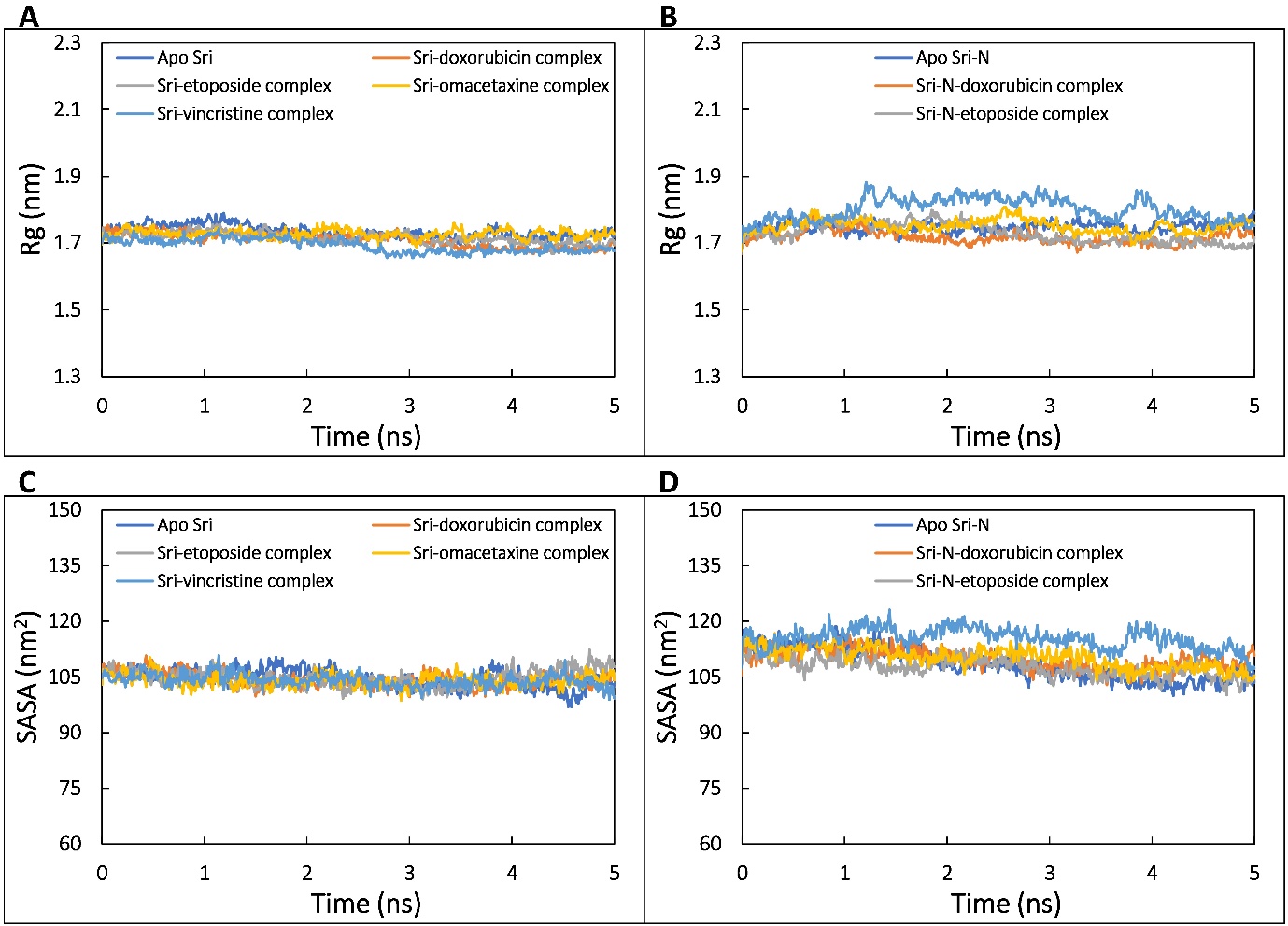


**Supplementary Fig S9.** **(A)** Radius of gyration (Rg) of the backbone atoms of the reported (Sri) in the absence and presence of ligands. **(B)** Radius of gyration (Rg) of the backbone atoms of the novel (Sri-N) in the absence and presence of ligands. **(C)** Solvent accessible surface area (SASA) of the backbone atoms of the reported (Sri) in the absence and presence of ligands. **(D)** Solvent accessible surface area (SASA) of the backbone atoms of the novel (Sri-N) in the absence and presence of ligands.


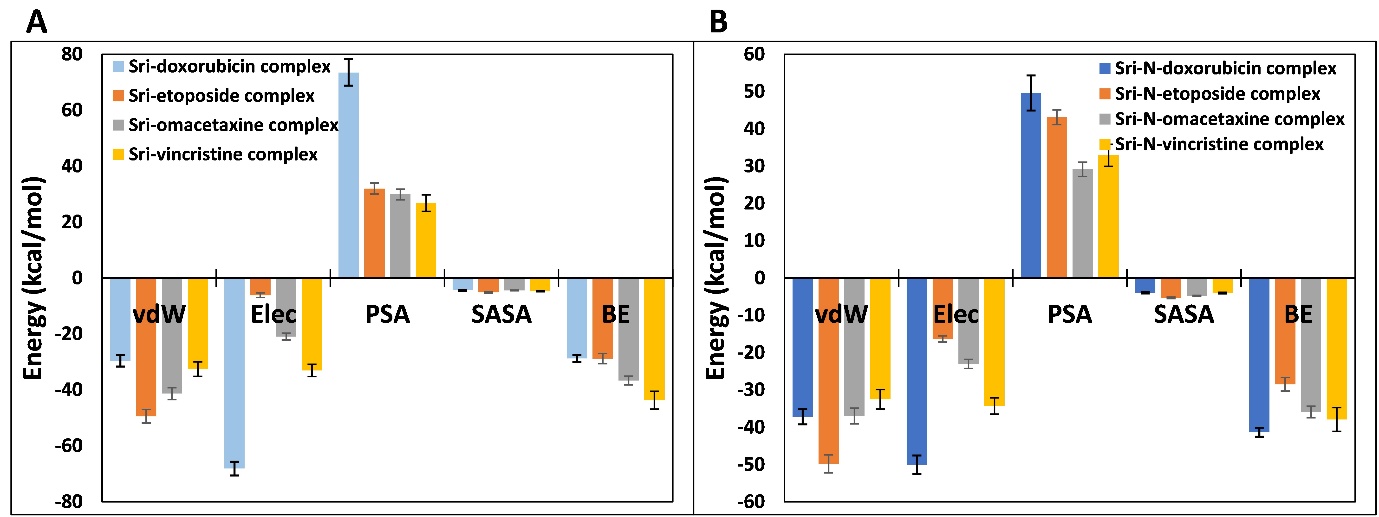


**Supplementary Fig S10.** **(A)** MM-PBSA binding energies for the interaction of ligands with Sri. **(B)** MM-PBSA binding energies for the interaction of ligands with Sri-N.


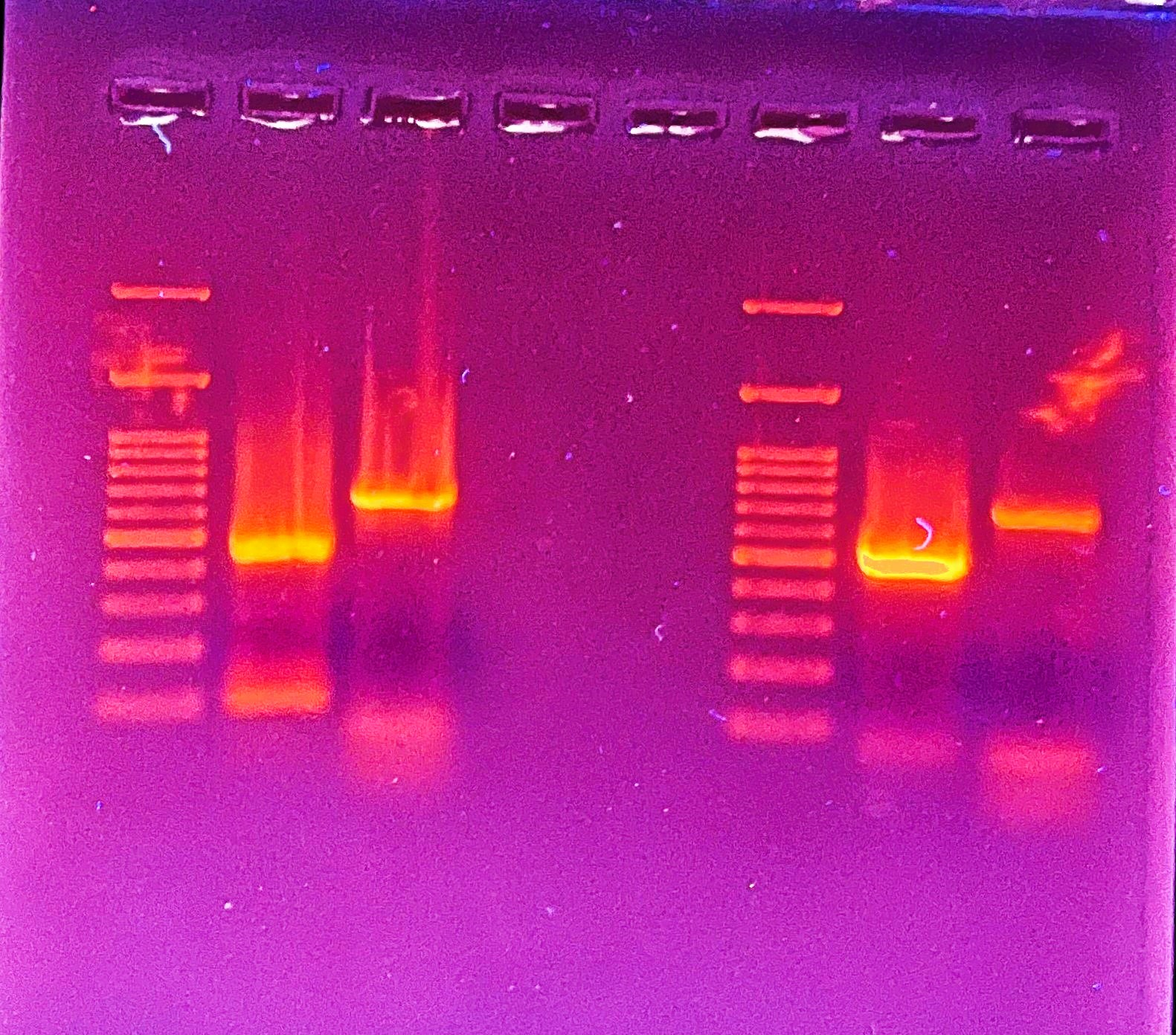


**Supplementary Figure S11.** Full length image of Agarose gel from where Figure 3A and 3B were cropped.

**
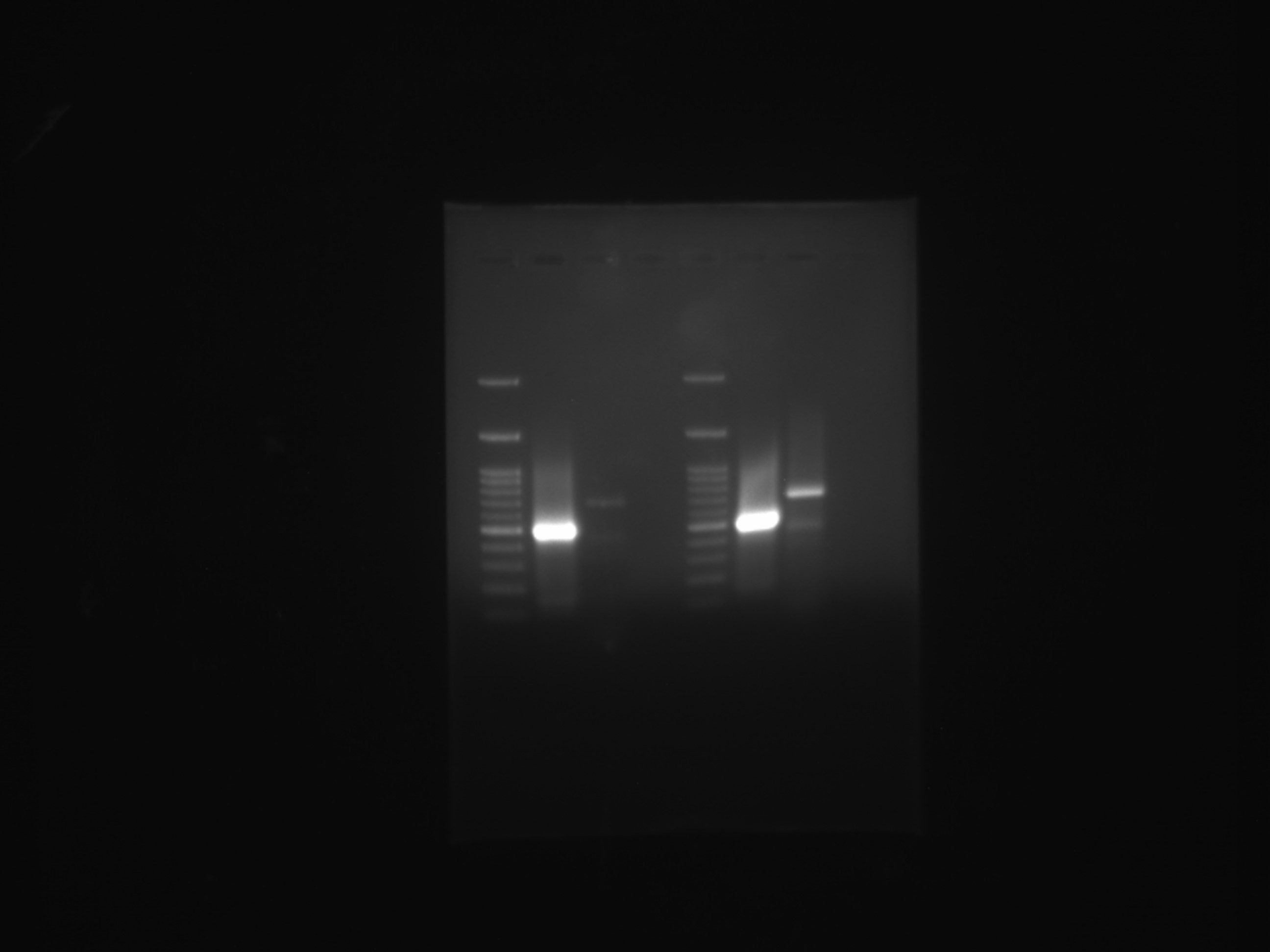
**

**Supplementary Figure S12.** Full length image of Agarose gel from where Figure 3C were cropped. Expression of Sri and Sri-N were checked in the liver.
